# CARD9-Dependent Neutrophil Recruitment Protects against Fungal Invasion of the Central Nervous System

**DOI:** 10.1371/journal.ppat.1005293

**Published:** 2015-12-17

**Authors:** Rebecca A. Drummond, Amanda L. Collar, Muthulekha Swamydas, Carlos A. Rodriguez, Jean K. Lim, Laura M. Mendez, Danielle L. Fink, Amy P. Hsu, Bing Zhai, Hatice Karauzum, Constantinos M. Mikelis, Stacey R. Rose, Elise M. N. Ferre, Lynne Yockey, Kimberly Lemberg, Hye Sun Kuehn, Sergio D. Rosenzweig, Xin Lin, Prashant Chittiboina, Sandip K. Datta, Thomas H. Belhorn, Eric T. Weimer, Michelle L. Hernandez, Tobias M. Hohl, Douglas B. Kuhns, Michail S. Lionakis

**Affiliations:** 1 Fungal Pathogenesis Unit, Laboratory of Clinical Infectious Diseases (LCID), National Institute of Allergy and Infectious Diseases (NIAID), National Institutes of Health (NIH), Bethesda, Maryland, United States of America; 2 Department of Microbiology, Icahn School of Medicine at Mount Sinai, New York, New York, United States of America; 3 Neutrophil Monitoring Laboratory, Applied/Developmental Research Directorate, Frederick National Laboratory for Cancer Research, Leidos Biomedical Research, Inc., Frederick, Maryland, United States of America; 4 Immunopathogenesis Section, LCID, NIAID, NIH, Bethesda, Maryland, United States of America; 5 Infectious Disease Service, Department of Medicine, Memorial Sloan-Kettering Cancer Center, New York, New York, United States of America; 6 Bacterial Pathogenesis Unit, LCID, NIAID, NIH, Bethesda, Maryland, United States of America; 7 Department of Biomedical Sciences, School of Pharmacy, Texas Tech University Health Sciences Center, Amarillo, Texas, United States of America; 8 Department of Laboratory Medicine, NIH Clinical Center, NIH, Bethesda, Maryland, United States of America; 9 Department of Molecular and Cellular Oncology, Division of Basic Science Research, The University of Texas MD Anderson Cancer Center, Houston, Texas, United States of America; 10 Surgical Neurology Branch, National Institute of Neurological Disorders and Stroke (NINDS), NIH, Bethesda, Maryland, United States of America; 11 Department of Pediatrics, University of North Carolina at Chapel Hill, Chapel Hill, North Carolina, United States of America; 12 Department of Pathology and Laboratory Medicine, University of North Carolina at Chapel Hill, Chapel Hill, North Carolina, United States of America; 13 Center for Environmental Medicine, Asthma, and Lung Biology, University of North Carolina at Chapel Hill, Chapel Hill, North Carolina, United States of America; University of Birmingham, UNITED KINGDOM

## Abstract

*Candida* is the most common human fungal pathogen and causes systemic infections that require neutrophils for effective host defense. Humans deficient in the C-type lectin pathway adaptor protein CARD9 develop spontaneous fungal disease that targets the central nervous system (CNS). However, how CARD9 promotes protective antifungal immunity in the CNS remains unclear. Here, we show that a patient with CARD9 deficiency had impaired neutrophil accumulation and induction of neutrophil-recruiting CXC chemokines in the cerebrospinal fluid despite uncontrolled CNS *Candida* infection. We phenocopied the human susceptibility in *Card9* ^*-/-*^ mice, which develop uncontrolled brain candidiasis with diminished neutrophil accumulation. The induction of neutrophil-recruiting CXC chemokines is significantly impaired in infected *Card9* ^*-/-*^ brains, from both myeloid and resident glial cellular sources, whereas cell-intrinsic neutrophil chemotaxis is Card9-independent. Taken together, our data highlight the critical role of CARD9-dependent neutrophil trafficking into the CNS and provide novel insight into the CNS fungal susceptibility of CARD9-deficient humans.

## Author Summary

CARD9 is a molecule expressed by mammalian immune cells and is centrally positioned downstream of several C-type lectin receptors, which sense fungi. The critical role of CARD9 in the activation of antifungal host defense has been highlighted by the demonstration that human mutations that disrupt the function of CARD9 are associated with the development of spontaneous life-threatening fungal infections, many of which target the central nervous system (CNS). However, why CARD9-deficient patients develop fungal disease in the CNS remained unclear. Here, we show that CARD9 is required for the recruitment of neutrophils, an immune cell critical for antifungal host defense, to the fungal-infected CNS in both mice and humans. Furthermore, we show that CARD9-deficient mice and humans are unable to produce the molecules (chemoattractants) needed to recruit neutrophils to the site of infection, thus identifying the mechanism for the striking absence of these cells in the infected CNS. Our work helps explain why CARD9 deficiency associates with CNS fungal disease, and furthers our understanding of how antifungal immunity operates in this complex tissue.

## Introduction

Human systemic fungal infections are typically opportunistic and primarily affect patients with acquired immunodeficiency (i.e. commonly due to HIV infection or modern medical interventions such as cancer chemotherapy and transplantation) or inborn errors of immunity [1, 2]. Systemic candidiasis, in particular, is the most common deep-seated fungal infection in the developed world and is now the most common cause of nosocomial bloodstream infection in the US [3]. Despite the availability of potent antifungal drugs, clinical outcomes of infected patients are often poor leading to significant morbidity and unacceptably high mortality rates [1]. Adjunctive immune-based therapies are therefore becoming increasingly desirable, yet their development and success depends on improving our understanding of the cellular and molecular basis of human antifungal immunity.

Fungi are recognized by the host immune system via innate pattern recognition receptors (PRRs) that are predominantly expressed by myeloid cells, including neutrophils, monocytes/macrophages and dendritic cells. C-type lectin receptors (CLRs) are a large class of PRRs of which several members including Dectin-1, Dectin-2 and Mincle have been shown to play crucial roles in antifungal immunity [4]. These receptors bind components of the fungal cell wall and this innate recognition event leads to the initiation of intracellular signaling cascades that stimulate cellular responses such as phagocytosis, induction of the respiratory burst, and production of pro-inflammatory chemokines/cytokines [4]. Although different CLRs exhibit independent functions, they utilize a common signaling pathway involving the kinase Syk and the signaling adaptor, CARD9 [5].

CARD9 is one of the most crucial mammalian antifungal immune molecules identified to date. Genetic deletion of *Card9* in mice results in defective pro-inflammatory cytokine production by myeloid cells and leads to a substantial increase in mortality following *C*. *albicans* challenge compared to WT mice [6]. CARD9 is also indispensable for antifungal defense in humans. Autosomal recessive CARD9 deficiency due to biallelic missense or nonsense *CARD9* mutations leads to profound defects in production of pro-inflammatory cytokines in response to fungal-specific stimuli, including GM-CSF, IL-1β, IL-6 and TNFα [7–14]. Other known CARD9-dependent functions in humans include CR3-dependent killing of unopsonized yeast by neutrophils [7, 14, 15], generation of neutrophilic myeloid-derived suppressor cells [16], and, in some patients, generation of Th17 cells [8, 11].

Along with loss of these CARD9-dependent functions, patients deficient in CARD9 are highly susceptible to spontaneous development of systemic fungal infections, predominantly caused by *Candida* species [7, 8], as well as severe cutaneous and subcutaneous infections caused by dermatophytes collectively known as deep dermatophytosis [13, 17]. Interestingly, a large proportion of CARD9-deficient patients develop systemic candidiasis that specifically targets the central nervous system (CNS) without persistent fungemia [7–9, 12, 18]. In patients with intact CARD9 signaling, systemic candidiasis is most often associated with the kidney, liver and spleen [19]. Therefore, this striking CNS-targeted manifestation of systemic candidiasis in CARD9-deficient patients suggests that CARD9 is uniquely required for protecting the CNS from fungal invasion. However, the CARD9-dependent immune effector functions operating in the CNS remain undefined.

Here, we describe a novel *CARD9* missense mutation (c.170G>A; p.R57H) identified in an 11 year-old girl with *Candida* meningoencephalitis. We extensively characterized the impact of this mutation on the antifungal host response in human myeloid cells, focusing on neutrophils, which are the primary immune effector cells against systemic *C*. *albicans* infections. Furthermore, we phenocopied the human CNS susceptibility to *C*. *albicans* infection in Card9 knockout (KO) mice, and characterized the Card9-dependent immune protective functions in this tissue *in vivo*. We have found that CARD9 is critical for control of fungal invasion in the CNS, acting to promote fungal-specific and tissue-specific neutrophil trafficking from the blood to the target organ via neutrophil-targeted chemokine production in both mice and humans.

## Results

### *Candida* Spinal Osteomyelitis and Meningoencephalitis in a Patient with a Novel Homozygous *CARD9* Missense Mutation

**Fig 1:**
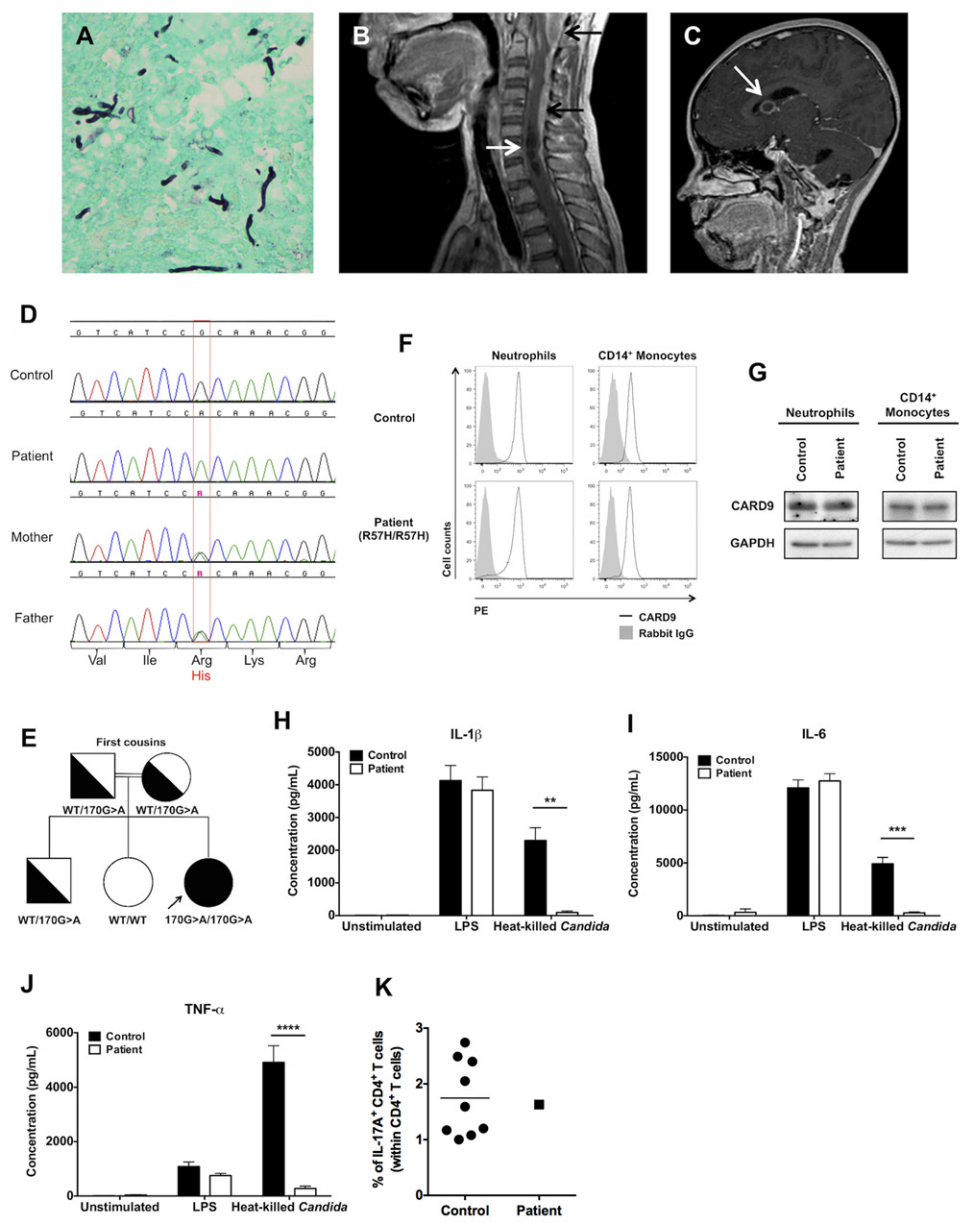
The autosomal recessive c.170G>A *CARD9* missense mutation results in impaired pro-inflammatory cytokine responses in PBMCs upon fungal stimulation. **(A)** Grocott-Gomori methenamine-silver stain of the patient’s T12 vertebral body biopsy sample showing filamentous fungal elements consistent with *C*. *albicans* that grew in culture (magnification, ×400). **(B)** Sagittal magnetic resonance image of the cervical spine showing leptomeningeal enhancement (black arrows) and a C5-T1 syrinx (white arrow). **(C)** Sagittal magnetic resonance image of the brain showing an abscess (arrow). **(D)** Sequencing chromatograms of *CARD9* at position 170 for the patient, her parents and a healthy donor control. **(E)** Pedigree of the patient with *Candida* meningoencephalitis. The patient is indicated in black and by arrow and the genotype of *CARD9* alleles is indicated below each family member. **(F)** FACS histograms of CARD9 staining in neutrophils and CD14^+^ monocytes of healthy donors and the patient (n = 2). **(G)** Representative Western blots of CARD9 protein expression in neutrophils and CD14^+^ monocytes of healthy donors and the patient. GAPDH is shown as loading control (n = 3). **(H-J)** IL-1β, IL-6 and TNFα production by healthy donor (n = 10) and patient (n = 4) PBMCs after 48 hours of stimulation with heat-killed *C*. *albicans* or LPS or after 48 hours without stimulation (n = 4–8 independent experiments). Data is analyzed by Mann Whitney U-test or unpaired t-test, where appropriate. **P < 0.01; ***P < 0.001. **(K)** Percentage of CD4^+^ IL-17A^+^ T cells (within CD4^+^ T-cells) of the patient and 9 healthy donors following stimulation with PMA/ionomycin, assessed by FACS. All quantitative data represent mean ± SEM.

An 11-year-old girl living in North Carolina in the US and born to consanguineous El Salvadorian parents presented at 8 years of age with fever, back pain, headache and vomiting and was diagnosed with T12-L1 spine osteomyelitis with associated diskitis and paraspinal abscess. She had recurrent oral thrush since birth but no other infections or medical conditions. Vertebral body bone biopsy showed granulomatous inflammation and necrosis with abundant *Candida* pseudohyphae (Fig 1A) and culture of the biopsy sample grew pan-sensitive *Candida albicans*. The patient received a 6-month course of fluconazole with clinical improvement. Approximately 15 months after discontinuation of fluconazole treatment, at the age of 9, the patient’s infection recurred and she presented with fever, neck and back pain, headache and vomiting. Cerebrospinal fluid (CSF) analysis, which was performed this time, revealed pleocytosis (213 nucleated cells/μl; 51% lymphocytes, 27% monocytes, 22% eosinophils), elevated protein (168 mg/dL) and decreased glucose (<20 mg/dL); CSF culture grew pan-sensitive *C*. *albicans* and beta-D-glucan (BDG) in the serum and CSF was markedly elevated at >500. Magnetic resonance imaging revealed cervical spine osteomyelitis, leptomeningeal enhancement (Fig 1B, black arrows) complicated by a large syrinx (Fig 1B, white arrow) and obstructive hydrocephalus that necessitated placement of a ventriculoperitoneal shunt, and brain abscesses (Fig 1C, arrow). No liver, splenic, intestinal or renal lesions were noted on abdominal imaging and no heart valve vegetation was seen by echocardiogram. Immunologic analyses of peripheral blood revealed normal monocyte, neutrophil, eosinophil, CD4^+^ T, CD8^+^ T, CD19^+^ B, and natural killer lymphocyte numbers. Phagocyte oxidative burst assessed by the dihydrorhodamine (DHR) assay was normal. T-lymphocyte proliferations were normal in response to PHA and PWM but decreased or absent in response to *Candida* and tetanus antigens. The levels of the patient’s IgA, IgE, IgG and IgM were normal. Neither her parents nor her siblings had a history of infections.

Since autosomal recessive CARD9 deficiency has been shown to predispose to *Candida* meningoencephalitis [7–9, 12], we sequenced CARD9 exons (S1 Table) and found a homozygous *CARD9* missense mutation, c.170G>A in exon 3 causing a substitution of histidine for arginine at position 57 (R57H), a highly conserved residue within the CARD domain of the CARD9 protein (Fig 1D and S1 Fig). The patient’s parents and brother are healthy and heterozygous for the mutation (WT/c.170G>A) whereas the patient’s healthy sister has WT CARD9 alleles (Fig 1D and 1E), consistent with autosomal recessive CARD9 deficiency with complete clinical penetrance. The c.170G>A mutation was not found in the 1000 Genomes or ExAC databases covering 117,000 chromosomes in the specific *CARD9* exon, thus this mutation is unlikely to be an irrelevant polymorphism. In addition, it was predicted to be deleterious by SIFT (deleterious), CADD [PHRED score 17.7] [20] and PolyPhen 2 [highest possible score of 1] [21]. Collectively, these data strongly suggest that the patient is homozygous for a rare and deleterious mutant *CARD9* allele.

### The c.170G>A *CARD9* Mutation Results in Generation of Full-Length CARD9 Protein with Impaired Function

Various *CARD9* mutations that lead to production of full-length, truncated or absent CARD9 proteins have been reported to date [7, 8, 12, 13]. Hence, we next investigated the impact of the c.170G>A mutation on CARD9 protein levels by FACS and Western blot analyses on the patient neutrophils and CD14^+^ monocytes and compared them to that of healthy donor myeloid cells. FACS revealed similar expression of CARD9 in WT and patient neutrophils and CD14^+^ monocytes (Fig 1F), whereas, as expected, no significant CARD9 expression was observed on T lymphocytes (S2 Fig). Similarly, immunoblot analyses for CARD9 on whole-cell extracts of the patient neutrophils and CD14^+^ monocytes demonstrated that CARD9 protein expression levels and molecular weight were similar with that of WT cells (Fig 1G). These data are in agreement with previous reports in which nonsense mutations prevented the generation of full-length CARD9 protein, whereas missense mutations did not predictably do so [7, 8, 12, 13].

We next sought to examine the functional consequences of the c.170G>A mutation by measuring the production of pro-inflammatory cytokines and chemokines from peripheral blood mononuclear cells (PBMCs) stimulated with either heat-killed *C*. *albicans* or LPS. Consistent with previously reported *CARD9* mutations [8, 12, 13], the c.170G>A mutation resulted in dramatically decreased IL-1β, IL-6 and TNF-α production after 48 hours of stimulation with *C*. *albicans* compared with 10 healthy donors, while cytokine production following LPS stimulation was comparable in the patient and healthy donor PBMCs (Fig 1H–1J). Furthermore, the production of GM-CSF, IFN-γ, IL-1α, IL-2, IL-10, CCL3, CCL4, CCL5, CCL7 and CXCL8 was also found to be CARD9-dependent in human PBMCs following fungal stimulation, whereas production of IL-4, IL-7, IL-15, IL-16, CCL2, CXCL1, CXCL2 and CXCL5 was CARD9-independent (S3 Fig). Since decreased proportions of IL-17^+^ CD4^+^ T cells have been reported in some, but not all, CARD9-deficient patients [7, 8, 10, 12, 13], we evaluated the production of IL-17A by the patient’s CD4^+^ T cells following PMA/ionomycin stimulation *ex vivo* using FACS, and found no impairment relative to 9 healthy donors (Fig 1K). Taken together, these data show that the c.170G>A mutation results in the generation of full-length CARD9 protein which exhibits impaired capacity for production of pro-inflammatory mediators upon fungal stimulation.

### Human Phagocytes Require CARD9 for Killing Fungal Yeasts but Not Hyphae, which Are Present in Infected Tissue

**Fig 2:**
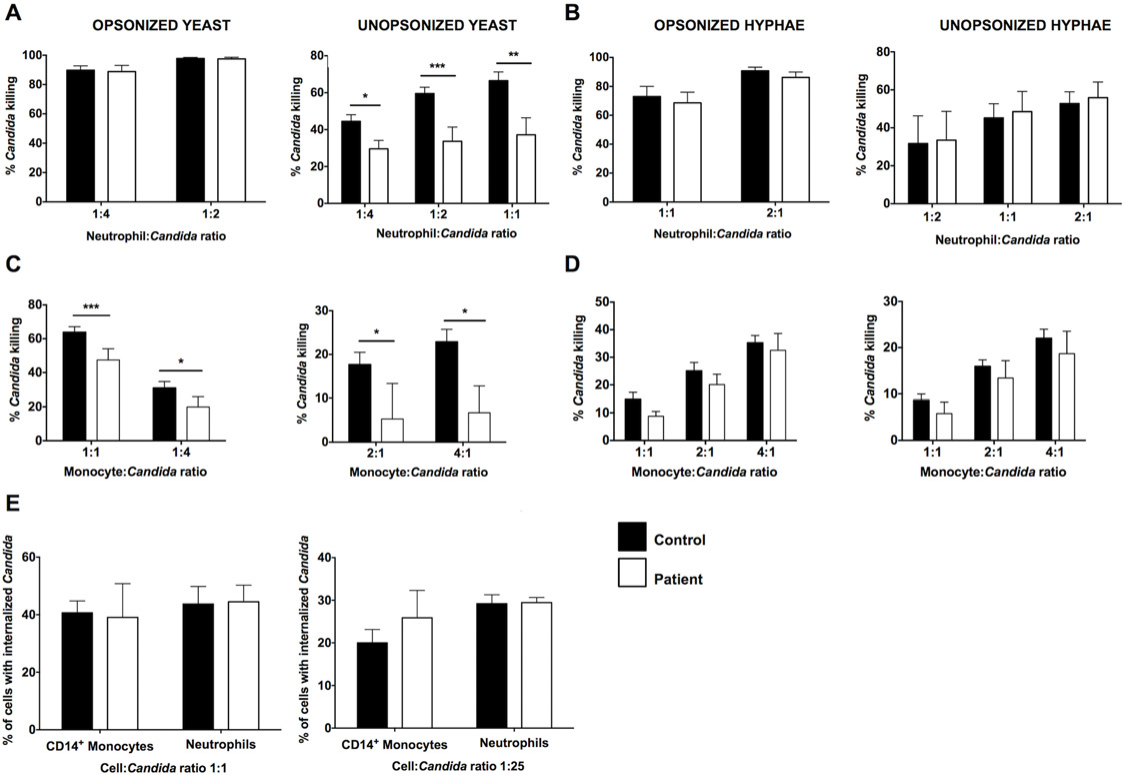
CARD9 mediates killing of *Candida* yeasts by human phagocytes but it is dispensable for the killing of invasive *Candida* hyphal forms that are found in infected tissue. Killing of opsonized and unopsonized *Candida* yeasts **(A)** and hyphal forms **(B)** by healthy donor and patient neutrophils (n = 5–7). Killing of opsonized and unopsonized *Candida* yeasts **(C)** and hyphal forms **(D)** by healthy donor and patient CD14^+^ monocytes (n = 5–8). **(E)** Internalization of opsonized (left panel) and unopsonized (right panel) *Candida* yeasts by healthy donor and patient neutrophils and CD14^+^ monocytes (n = 3 independent experiments). Data is analyzed by Mann Whitney U-test or unpaired t-test, where appropriate. *P < 0.05; **P = 0.01; ***P < 0.001. All quantitative data represent mean ± SEM.

Neutrophils and monocytes/macrophages are the critical cellular mediators of host defense against systemic *C*. *albicans* infection [19, 22, 23]. Therefore, we next investigated the effects of the c.170G>A mutation on the ability of neutrophils and monocytes to kill the two major morphological forms of *C*. *albicans*, yeasts and hyphae. Hyphae are the invasive filamentous fungal forms present in human infected CNS tissue (Fig 1A). It has been previously shown that CARD9 deficiency impairs the killing capacity of neutrophils against unopsonized, but not opsonized, *C*. *albicans* yeasts [7, 15]. Indeed, we found decreased killing by the patient’s neutrophils of unopsonized *C*. *albicans* yeasts whereas neutrophil killing was intact against opsonized yeasts (Fig 2A). However, CARD9-deficient neutrophils did not exhibit impaired killing capacity against opsonized or unopsonized *C*. *albicans* hyphae (Fig 2B), which are the predominant form found in infected CNS tissue of mice and humans (Fig 1A) [24, 25]. We then examined the impact of CARD9 deficiency on the ability of CD14^+^ monocytes to kill *C*. *albicans* yeasts and hyphae. CD14^+^ monocytes demonstrated inferior killing capacity of *C*. *albicans* relative to neutrophils (Fig 2A–2D). As shown in Fig 2C and 2D, CD14^+^ monocytes from the patient exhibited a modest decrease in killing of *C*. *albicans* yeasts, but hyphal killing was intact. The decreases observed in killing of yeasts by the patient’s neutrophils and CD14^+^ monocytes were not due to impaired fungal internalization (Fig 2E). Collectively, these data show that although CARD9-deficient neutrophils have a decreased ability to kill unopsonized *C*. *albicans* yeasts, CARD9 is dispensable for killing of the invasive hyphal forms found in infected CNS tissue. Therefore, other factors in addition to CARD9-mediated *C*. *albicans* yeast killing by phagocytes are likely to also contribute to the susceptibility of CARD9-deficient patients to *Candida* infection of the CNS.

### CARD9 Deficiency Results in a Striking Absence of Neutrophils in the Infected CNS

**Fig 3:**
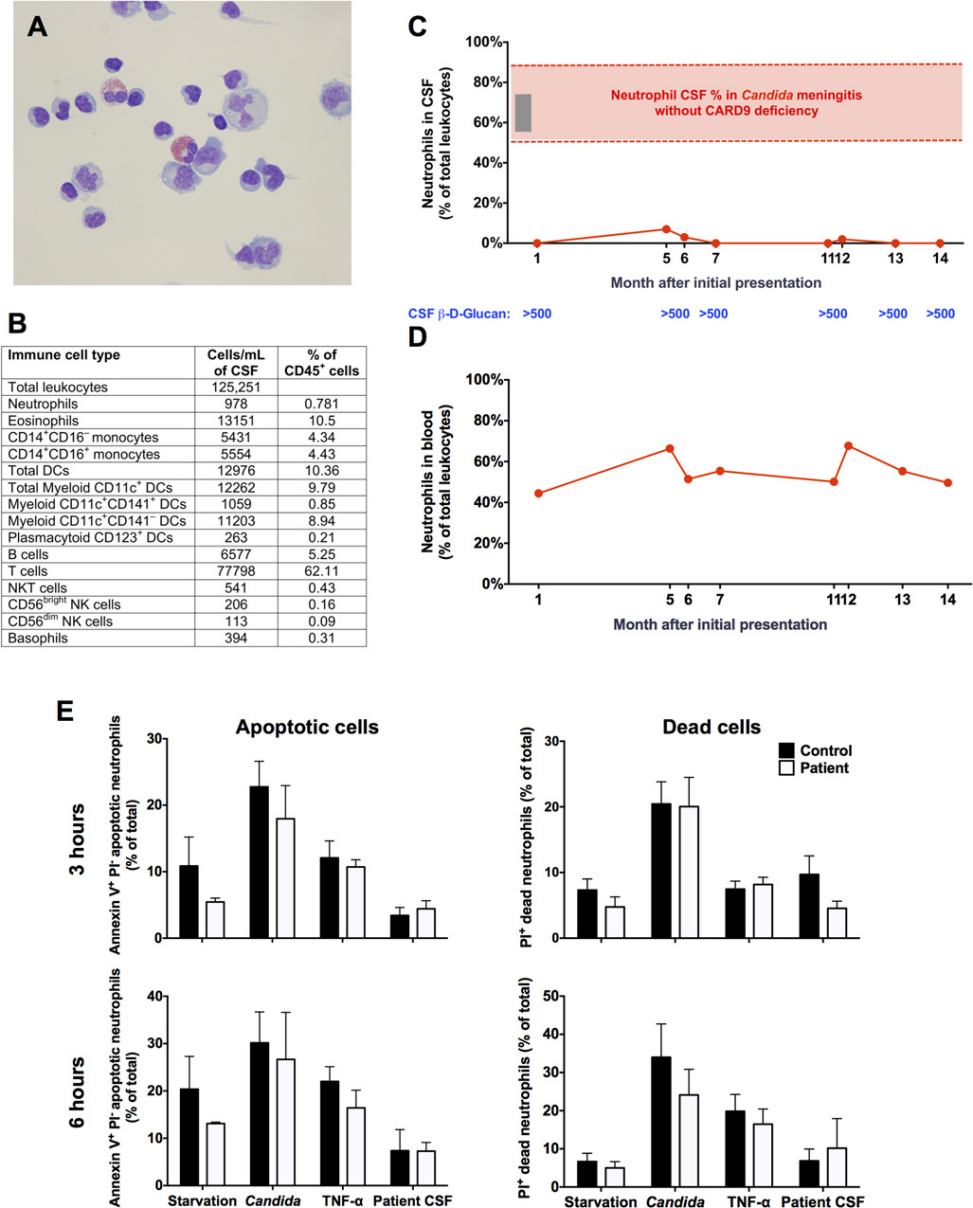
CARD9 deficiency results in a striking absence of neutrophils from the infected CSF that is not caused by peripheral neutropenia or decreased neutrophil survival. **(A)** Representative cytopathology image of the patient’s infected CSF showing lymphocytes, eosinophils and mononuclear phagocytes, but no neutrophils. **(B)** Immunophenotype of the patient’s infected CSF, assessed by FACS. **(C)** Percentages of neutrophils within leukocytes in the CARD9-deficient patient’s infected CSF over time. The frequency range of neutrophils in the CSF found in our CARD9^+/+^ patient with *Candida* meningitis post-Ommaya reservoir placement (grey bar) and those typically reported for *Candida* meningoencephalitis in patients without CARD9 deficiency (red bar) are also shown. **(D)** The percentages of neutrophils within leukocytes in peripheral blood of the CARD9-deficient patient over time. **(E)** Healthy donor and patient neutrophils were cultured for 3 hours (upper panels) or 6 hours (lower panels) under the indicated conditions and FACS used to assess the percentage of Annexin V^+^ PI^-^ apoptotic (left panels) and Annexin V^+^ PI^+^ dead (right panels) cells (n = 3–4; 3 independent experiments). All quantitative data represent mean ± SEM.

Notably, we observed that the patient had a striking absence of neutrophils in the infected CSF, despite uncontrolled fungal disease. Fig 3A shows a representative cytopathology image of the infected CSF, in which lymphocytes, eosinophils and mononuclear phagocytes could be found, but neutrophils were absent. Indeed, FACS analysis of the infected CSF revealed that the predominant accumulating leukocytes were T-lymphocytes and eosinophils (Fig 3B), which are not critical mediators of host defense against systemic *C*. *albicans* infection [19]. This finding is in agreement with other reports of high numbers and predominance of lymphocytes and eosinophils in the CSF of *Candida*-infected CARD9-deficient patients [7, 9]. Monocytes, dendritic cells and B-cells were also present (5–10% each), whereas neutrophils comprised <1% of total leukocytes (Fig 3B). Over time, the lack of neutrophils was consistent with these cells never exceeding 7% of total leukocytes in the infected CSF (range: 0–7%) despite continued inflammation (range of nucleated cells in CSF: 108-422/μl; range of % of eosinophils within leukocytes in CSF: 3–40%; range of lymphocytes within leukocytes in CSF: 25–72%; range of protein levels in CSF: 161–845 mg/dL) and extremely high fungal load indicated by persistently elevated CSF BDG (Fig 3C). Persistent *Candida* tissue hyphal invasion and absence of neutrophil infiltration were confirmed in the patient’s CNS tissue by brain biopsy (S4 Fig). The decreased neutrophil accumulation noted in our patient is grossly suboptimal compared to the robust neutrophil accumulation seen in patients without CARD9 deficiency when they develop *C*. *albicans* meningitis, typically post-neurosurgical procedures (Fig 3C; red bar) [26–29]. Consistent with that, a patient at NIH who developed *C*. *albicans* meningitis post-Ommaya reservoir placement exhibited pleiocytosis (133–1091 nucleated cells/μl) and significant neutrophil mobilization in the infected CSF (56–73% of total leukocytes) (Fig 3C; grey bar).

We next sought to investigate the etiology of suboptimal neutrophil accumulation in the infected CSF in the setting of CARD9 deficiency. The patient did not manifest peripheral neutropenia (range of absolute neutrophil count in the blood: 1,960–5,340/mm^3^), ruling out this possibility (Fig 3D). We next examined whether CARD9 is a survival factor for human neutrophils. For that, we harvested neutrophils from the peripheral blood of the patient and healthy donors and determined differential cell apoptosis and death at 3 and 6 hours after serum starvation, TNF-α stimulation or live *C*. *albicans* challenge *ex vivo*. Neutrophils were also directly exposed to the patient’s infected CSF to evaluate for the presence of potential soluble pro-apoptotic factors within the infected tissue. We found that CARD9-deficient neutrophils did not exhibit enhanced apoptosis or death under any of the tested conditions relative to WT neutrophils (Fig 3E). Therefore, these data demonstrate a striking absence of neutrophil accumulation in the *Candida*-infected CSF in human CARD9 deficiency, which is not caused by peripheral neutropenia or defective neutrophil survival.

### CARD9 Is Critical for the Production of Neutrophil-Targeted Chemoattractants in the Infected CSF

**Fig 4:**
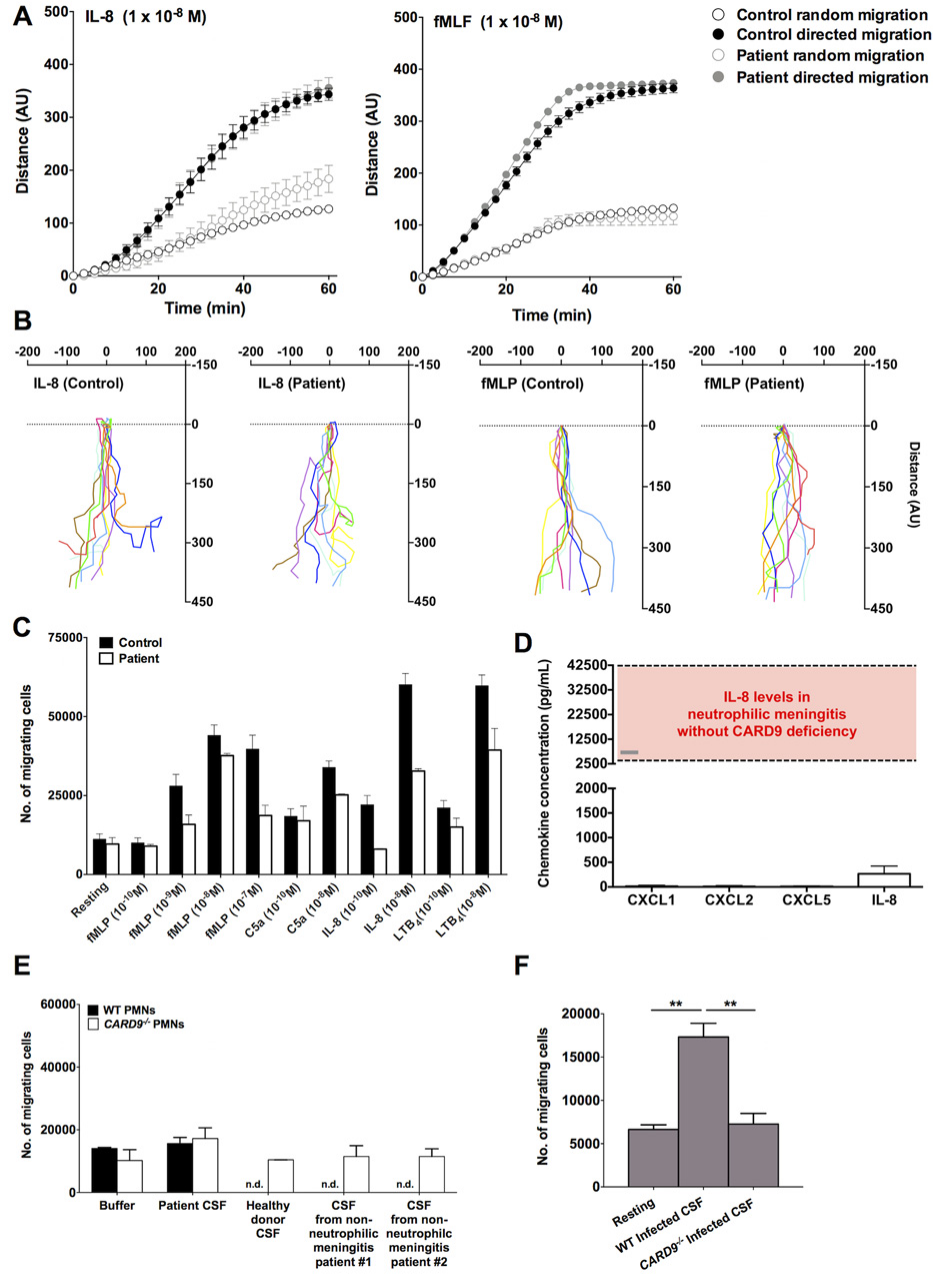
CARD9 is critical for the generation of neutrophil-targeted chemokines in the infected CSF but it is dispensable for intrinsic chemotaxis of human neutrophils. **(A)** Pooled data showing chemotaxis of healthy donor (n = 40) and patient (n = 2) neutrophils towards IL-8 (left panel) and fMLP (right panel) assessed by the EZ-TAXIScan system. **(B)** EZ-TAXIScan data for individual neutrophils moving towards either IL-8 (left panels) or fMLP (right panels); individual tracks are represented by different colours and were recorded over 1 hour. **(C)** Chemotaxis of healthy donor (n = 13–15) and patient (n = 2) neutrophils towards fMLP, C5a, IL-8 and LTB4 at the indicated concentrations, assessed by the 96-well Neuroprobe chamber. Data is analyzed by two-way ANOVA (with Bonferroni correction). **(D)** Levels of neutrophil-targeted chemokines in the CARD9-deficient patient’s infected CSF (n = 3). The range of IL-8 in the CSF found in our CARD9^+/+^ patient with *Candida* meningitis post-Ommaya reservoir placement (grey bar) and previously reported for neutrophilic meningitis in patients without CARD9 deficiency (red bar) are also shown. **(E)** The patient’s infected CSF is not chemotactic toward healthy donor and patient neutrophils, as assessed by the 96-well Neuroprobe chamber (n = 2). Resting indicates neutrophil migration toward buffer; nd: not detected. **(F)** The CSF of the CARD9^+/+^ *Candida*-infected patient post-Ommaya reservoir, but not the CSF of the CARD9-deficient patient, is chemotactic toward healthy donor neutrophils, as assessed by the 96-well Neuroprobe chamber (n = 2–4). Resting indicates neutrophil migration toward buffer. **P < 0.01 by unpaired t-tests. All quantitative data represent mean ± SEM. fMLP, formyl-methionyl-leucyl-phenylalanine; LTB4, leukotriene B4; PMNs, neutrophils.

We reasoned that the decreased neutrophil recruitment in the CNS of the patient could be explained by the net effect of (1) impaired intrinsic chemotactic ability of CARD9-deficient human neutrophils and/or (2) defective production of neutrophil-targeted chemoattractants in the infected tissue. Thus, we tested whether CARD9-deficient neutrophils have cell-intrinsic chemotactic defects and/or whether the neutrophil-targeted chemoattractants are not produced in order to generate the chemotactic gradient that is necessary for cell trafficking into the infected CNS. We first assessed neutrophil chemotaxis using both the EZ-TAXIScan system and a modified Boyden chamber, the 96-well Neuroprobe chamber. The EZ-TAXIScan instrument allows for simultaneous measurement of cell velocity and directionality by observing the spatial and temporal movement of individual cells toward a chemoattractant. Using this method, we found no defect in chemotaxis of CARD9-deficient neutrophils toward IL-8 or formyl-methionyl-leucyl-phenylalanine (fMLP) (Fig 4A and 4B), two prototypic chemotactic factors for human neutrophils. We then used the 96-well Neuroprobe chamber and examined neutrophil chemotaxis toward IL-8, fMLP, the leukotriene B4 (LTB4), and the complement factor C5a. Similar to our EZ-TAXIScan data, no impairment was found in the chemotaxis of CARD9-deficient neutrophils relative to WT neutrophils using the 96-well Neuroprobe chamber when correcting for multiple comparisons (Fig 4C).

Since the patient’s neutrophils did not exhibit a primary chemotaxis defect, we next measured levels of the neutrophil-targeted chemokines CXCL1, CXCL2, CXCL5 and IL-8 in the infected CSF by Luminex array at different prospective time-points during uncontrolled fungal infection. Strikingly, we found absent to very low levels of these chemokines (Fig 4D). IL-8 was detected at higher levels (mean: 267.6 pg/mL) than all other chemokines (mean: 13.3 pg/mL for CXCL1, 10.6 pg/mL for CXCL2, 16.3 pg/mL for CXCL5), but these IL-8 levels were comparable to previously reported IL-8 levels in uninfected CSF from healthy donors [30]. Interestingly, the measured levels of IL-8 are significantly lower than those detected in patients with neutrophilic meningitis in the absence of CARD9 deficiency (Fig 4D; red bar) [30]. Consistent with the absence of induction of neutrophil-targeted chemokines, CSF from the infected patient did not exhibit any greater chemotactic activity toward WT and patient neutrophils than that observed in buffer, CSF from a healthy subject or CSF from two patients with lymphocytic (non-neutrophilic) meningitis (Fig 4E).

Instead, our CARD9^+/+^ patient who developed *C*. *albicans* meningitis post-Ommaya reservoir placement associated with neutrophil mobilization in the CSF exhibited significant induction of CXC neutrophil-recruiting chemokines in the infected CSF (Fig 4D; grey bar). Specifically, the CSF levels of IL-8, CXCL1, CXCL2 and CXCL5 were 17.1–fold (4576.8 pg/mL), 294.4–fold (3120.6 pg/mL), 16–fold (261.2 pg/mL) and 93.4–fold (1523.1 pg/mL) greater, respectively, relative to those detected in the CSF of our CARD9-deficient patient. In line with the induction of neutrophil-targeted CXC chemokines, *Candida*-infected CSF from the CARD9^+/+^ patient exhibited chemotactic activity toward WT neutrophils *ex vivo* (Fig 4F). Altogether, these data suggest that the absence of neutrophil accumulation in the infected CNS despite uncontrolled fungal disease correlates with a defect at the level of chemoattractant production in the infected tissue, whereas CARD9 is dispensable for cell-intrinsic neutrophil chemotaxis.

### Neutrophils Are Critical for Control of Fungal CNS Infection in Wild-Type (WT) Mice

**Fig 5:**
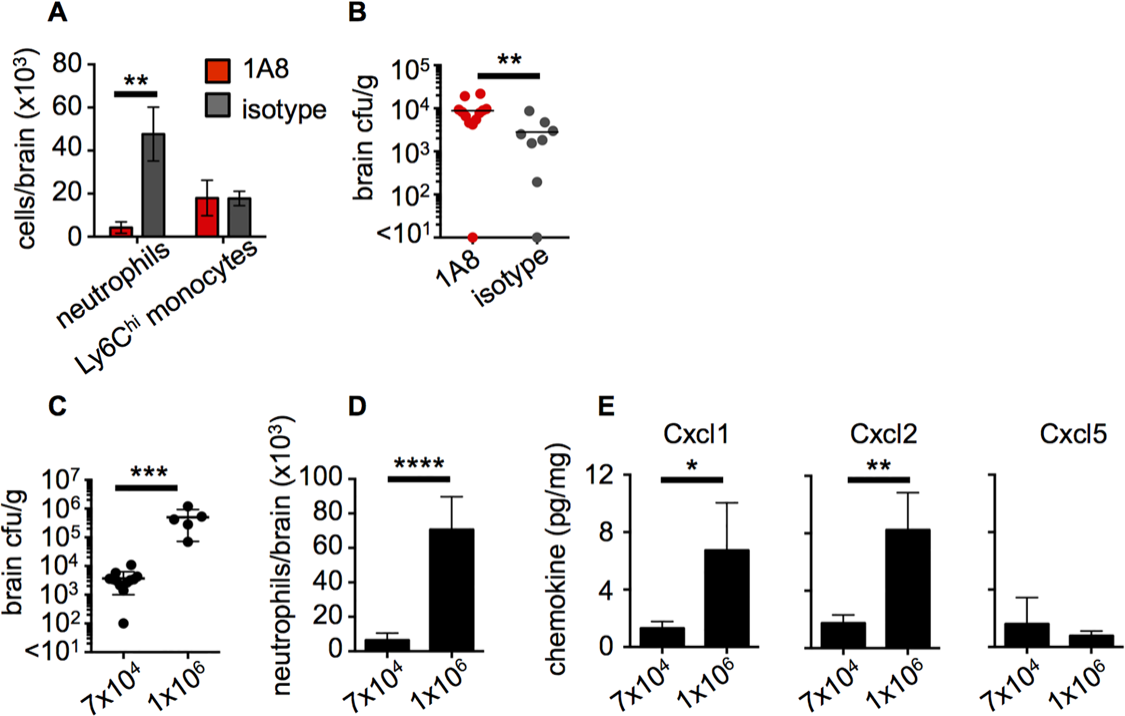
Neutrophil recruitment is critical for control of fungal brain infection and directly correlates with the extent of brain fungal burden in mice. WT animals were injected intravenously with 100 μg of 1A8 antibody (red bars, n = 10) or 2A3 isotype control antibody (grey bars, n = 8) at 24 hours prior to infection and at the time of infection. At 24 hours post-infection, brains from these mice were analyzed for (**A**) recruitment of myeloid cells by FACS and (**B**) brain fungal burden. Data pooled from two independent experiments and analyzed by two-way ANOVA (**A**) or Mann Whitney U-test (**B**). WT animals were systemically infected with a low (7x10^4^) or a high inoculum (1x10^6^) of *C*. *albicans* SC5314 and brains assessed for (**C**) fungal burden, (**D**) neutrophil recruitment by FACS and (**E**) production of neutrophil-targeted chemokines by Luminex array at 72 hours post-infection. Data shown is representative of 2 independent experiments and analyzed by unpaired t-tests (**C-E**). *P<0.05, **P<0.01, ***P<0.005, ****P<0.001. Data represent mean ± SEM.

To further examine the mechanistic role of CARD9 in antifungal defense of the CNS, we utilized a well-established mouse model of systemic *C*. *albicans* infection. This model results in fungal dissemination to the brain associated with an early influx of neutrophils, whose accumulation decreases later on as the infection is controlled [24]. To directly examine whether the recruitment of neutrophils to the CNS is critical for control of fungal proliferation in this tissue, we depleted neutrophils in WT mice using the 1A8 clone of the anti-Ly6G antibody [31]. We found that administration of 1A8 successfully depleted >90% of neutrophils in the brain of infected mice compared to infected mice treated with an isotype control antibody, and had no effect on the recruitment of other myeloid cells such as Ly6C^hi^ monocytes (Fig 5A). Analysis of brain fungal burdens in these animals revealed that neutrophil depletion by 1A8 led to a significant increase in brain fungal load compared to control animals (Fig 5B). Therefore, these data demonstrate that neutrophil recruitment to the CNS post-infection is critical for control of fungal brain infection.

### Fungal Infection of WT Mouse CNS Results in Chemokine Production and Neutrophil Recruitment That Correlate with the Extent of Brain Fungal Burden

We further analyzed the fungal-load dependence of the neutrophil response in the infected WT mouse CNS by infecting WT mice with either a low (7x10^4^) or a high (1x10^6^) inoculum of *C*. *albicans*. WT mice infected with the high inoculum developed significantly greater brain fungal burden compared to those infected with the low inoculum (Fig 5C). Using FACS, we assessed the number of recruited neutrophils in the brains of animals with high or low tissue burden. We found that animals infected with the high inoculum, which developed high brain fungal proliferation, had nearly 10-fold greater neutrophil numbers in the brain than observed in the mice which received the low inoculum and had lower tissue fungal burden (Fig 5D). Accordingly, we found that the levels of the major mouse neutrophil-targeted chemokines Cxcl1 (KC) and Cxcl2 (MIP-2) were significantly increased in the brains of mice with the higher tissue fungal burden (Fig 5E). In contrast, LPS-induced chemokine (LIX), the mouse homologue of CXCL5 which can also recruit neutrophils, was similar in all brains analyzed and had no correlation to the extent of brain fungal burden (Fig 5E), suggesting that Cxcl5 induction is not fungal-load dependent in the infected mouse brain. Taken together, these data show that neutrophil-targeted chemokine production and neutrophil mobilization to the infected CNS directly correlates with the extent of tissue fungal burden.

### Card9 Is Required for CNS Neutrophil Accumulation and Control of Fungal Brain Infection

**Fig 6:**
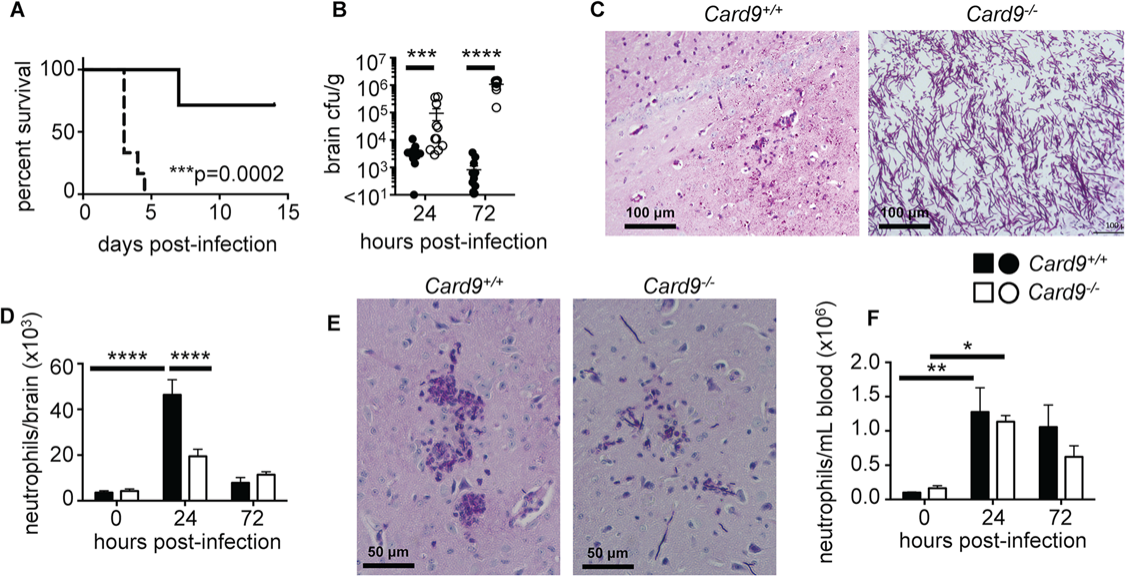
*Card9* ^*-/-*^ mice are highly susceptible to candidiasis of the CNS and do not accumulate neutrophils into the infected brain. (**A**) WT (solid line, n = 7) and *Card9* ^*-/-*^ (dotted line, n = 6) littermates were infected with 1x10^5^ CFU SC5314 *C*. *albicans* SC5314 yeasts intravenously and survival monitored for 14 days post-infection; data analyzed by log-rank (Mantel-Cox) test. (**B**) Brains were isolated from WT (filled circles) and *Card9* ^-/-^ (empty circles) mice at 24 (WT n = 12, KO n = 11) and 72 (WT n = 12, KO n = 10) hours post-infection and analyzed for fungal burden; data analyzed by Mann Whitney U-test. (**C**) PAS-stained brain sections from WT and *Card9* ^*-/-*^ mice at 72 hours post-infection. Images were taken in the cerebrum region of the brain. Images are representative of 6 animals from 2 independent experiments. (**D**) Neutrophil numbers were determined by FACS in the brain of WT (filled bars, 0 hours n = 5, 24 hours n = 6–13, 72 hours n = 6–13) and *Card9* ^-/-^ (empty bars, 0 hours n = 5–6, 24 hours n = 6–14, 72 hours n = 6–12) mice; data analyzed by two-way ANOVA. (**E**) PAS-stained brain sections from WT and *Card9* ^*-/-*^ mice at 24 hours post-infection, analyzed as described in (**C**). (**F**) Neutrophil numbers in the peripheral blood, analyzed as in (**D**). In B-F, mice analyzed at 24 hours were infected with 1.3x10^5^ CFU SC5314, and mice analyzed at 72 hours were infected with 7x10^4^ CFU. All data is pooled from 2–4 independent experiments; data represent mean ± SEM. ***P<0.005, ****P<0.001.

We next sought to understand the functional role of Card9 in the mouse brain during *C*. *albicans* infection. For this, we used *Card9* ^*-/-*^ mice that are significantly more susceptible to systemic candidiasis (Fig 6A), as has been previously shown [6]. Importantly, we phenocopied for the first time the human CNS susceptibility to *Candida* infection of CARD9-deficient patients in *Card9* ^*-/-*^ mice which developed uncontrolled brain infection, evidenced by significantly higher brain fungal burdens as early as 24 hours post-infection (Fig 6B). At 72 hours post-infection, *Card9* ^*-/-*^ mice exhibited dramatically greater brain fungal burdens (over 1000-fold) compared to WT animals. In agreement, histopathological analysis of infected brains revealed extensive tissue invasion in the *Card9* ^*-/-*^ brain by hyphae, which were the predominant fungal morphology observed in the infected brain (Fig 6C).

Since our CARD9-deficient patient had limited numbers of neutrophils in the infected CSF, we next investigated whether the same defect was present in the infected *Card9* ^*-/-*^ mouse brain. Indeed, we found a profound decrease in neutrophil accumulation in the *Card9* ^*-/-*^ brain at 24 hours post-infection compared with WT animals (Fig 6D and S5A Fig). Notably, while WT mice recruited high numbers of neutrophils to the brain early after infection, there was no significant increase in neutrophil numbers or frequency between uninfected and infected *Card9* ^*-/-*^ brains at any time point after infection, despite dramatic tissue fungal proliferation (Fig 6D and S5A Fig). Indeed, histopathology revealed that neutrophils could be seen to crowd around hyphae in WT brains at 24 hours post-infection, whereas very few of these cells were observed in *Card9* ^*-/-*^ brains (Fig 6E). *Card9* ^*-/-*^ brains had similar neutrophil numbers with WT brains at 72 hours post-infection; strikingly, these numbers were similar to those observed in the uninfected brain (Fig 6D). This reflects a markedly defective response in *Card9* ^*-/-*^ mice since these animals have over 1000-fold increase in brain fungal burden compared to WT (Fig 6B); hence, based on the inoculum-dependence of neutrophil accumulation in the mouse brain (Fig 5), these animals should have mobilized significantly more neutrophils into this tissue. In our patient and in other reported patients’ CSF [7, 9], in addition to a lack of neutrophils, we and others also observed high numbers of lymphocytes and eosinophils. To investigate if this was also true in *Card9* ^*-/-*^ mice, we calculated the relative frequency of multiple lymphoid and myeloid cell populations in the brain following infection by FACS. Besides a significant reduction in neutrophils and a corresponding increase in microglia percentages in *Card9* ^*-/-*^ brains, we did not find any other significant differences in the frequency of other leukocyte populations between WT and *Card9* ^*-/-*^ brains at the tested time points (S5B Fig). Collectively, these data show that, consonant to human findings, Card9 is required for the control of *C*. *albicans* infection of the CNS and for promoting neutrophil accumulation in the infected brain.

### Card9 Mediates Tissue-Specific and Fungal-Specific Neutrophil Accumulation into the Infected CNS

We next investigated whether neutrophil accumulation into the *C*. *albicans*-infected kidney is Card9-dependent. We found that, in line with previously reported findings [6], *Card9* ^*-/-*^ mice had significantly increased kidney fungal burdens at both 24 and 72 hours post-infection (S6A Fig). However, unlike the brain, *Card9* ^*-/-*^ mice were able to mobilize significant numbers of neutrophils into the kidney at 72 hours post-infection, approximately 3-fold greater than in WT infected kidneys at the same time-point post-infection, and nearly 100-fold greater than in uninfected control kidneys (S6B Fig). Although the extent of neutrophil accumulation may still be to a degree suboptimal relative to the higher kidney fungal load seen in *Card9* ^*-/-*^ kidneys [22, 24, 32], the data collectively indicate that Card9 plays organ-specific roles in neutrophil accumulation after systemic candidiasis, in agreement with a recent report showing neutrophil mobilization into the *C*. *tropicalis* infected *Card9* ^*-/-*^ kidney [33].

We then assessed whether the defect in neutrophil accumulation into the infected brain was specific to fungi or could be recapitulated with non-fungal pathogens. For this, we infected WT and *Card9* ^*-/-*^ animals intravenously with *Staphylococcus aureus*, an important human bacterial pathogen. We found that *Card9* ^*-/-*^ mice had similar bacterial burdens and accumulation of neutrophils in the brain compared to WT mice at 48 hours post-infection (S7 Fig). These data are in agreement with the lack of development of bacterial CNS infections in patients with CARD9 deficiency and show that the neutrophil accumulation defect we observed during *C*. *albicans* infection is specific for fungal pathogens.

### Card9 Is Not Required for Neutrophil Production and Egress from Bone Marrow

We next began to investigate possible mechanisms of the Card9-dependent accumulation of neutrophils in the brains of *C*. *albicans* infected mice. We first assessed whether Card9 is critical for the production of neutrophil progenitors in the bone marrow and egress of these cells into the peripheral blood after infection. We found that *C*. *albicans* infection led to a significant increase in neutrophil progenitors in the bone marrow (S8 Fig) and a significant induction of neutrophilia in the blood of WT mice (Fig 6F). No differences were observed in neutrophil numbers in the bone marrow (S8 Fig) and peripheral blood (Fig 6F) between WT and *Card9* ^*-/-*^ animals, either at steady state or after infection. Since neutrophil production and mobilization of large numbers of neutrophils from the bone marrow into the blood of *Card9* ^*-/-*^ mice following *C*. *albicans* infection did not translate into accumulation of these cells into the infected CNS (Fig 6D–6F), these data collectively suggest that Card9 is critical for the trafficking of neutrophils from the blood into the infected CNS.

### Card9^-/-^ Neutrophils Exhibit Normal Chemotaxis, Adhesion and Survival In Vivo

**Fig 7:**
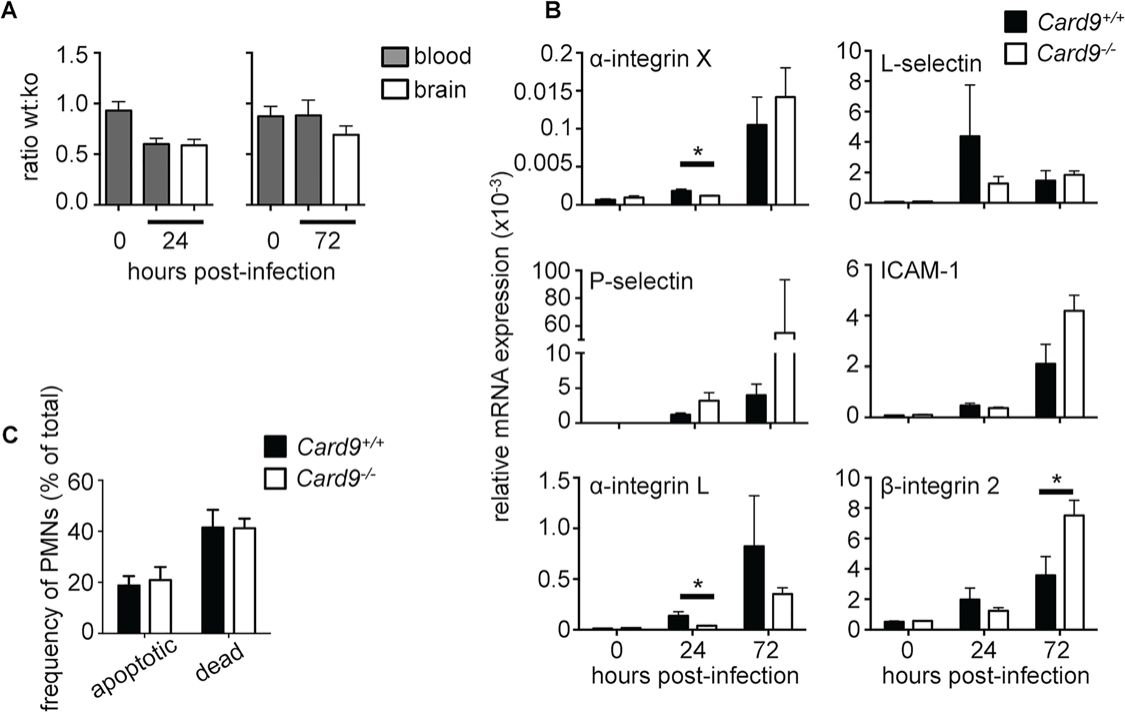
CARD9 deficiency does not affect cell-intrinsic neutrophil chemotaxis, survival or expression of adhesion molecules. **(A**) The ratio of CD45.1^+^ WT neutrophils to CD45.2^+^ *Card9* ^*-/-*^ neutrophils in mixed bone marrow chimeras was determined by FACS in the blood prior to infection and in the blood and brain at 24 (left panel) and 72 hours (right panel) post-infection (n = 7–8 per time point). (**B**) Expression of various adhesion molecules in whole brain homogenates of uninfected and infected WT (filled bars, n = 6) and *Card9* ^*-/-*^ (empty bars, n = 6) mice, as determined by qRT-PCR. Gene expression was calculated relative to Gapdh. Mice analyzed at 24 hours were infected with 1.3x10^5^ CFU SC5314, and mice analyzed at 72 hours infected with 7x10^4^ CFU. (**C**) Neutrophil survival in the brain of 24-hour infected (dose: 1.3x10^5^ CFU) WT (filled bars, n = 6) and *Card9* ^*-/-*^ (empty bars, n = 6) mice was determined by Annexin-V and 7-AAD staining and analysis by FACS. Apoptotic neutrophils were defined as Annexin-V^+^ 7-AAD^-^, while dead neutrophils were defined as Annexin-V^+^ 7-AAD^+^. All data is pooled from two independent experiments; data represent mean ± SEM. *P<0.05 by two-way ANOVA.

We showed earlier that CARD9-deficient human neutrophils had normal chemotaxis toward four major neutrophil-targeted chemoattractants *ex vivo* (Fig 4A–4C). To analyze the recruitment of WT and *Card9* ^*-/-*^ neutrophils into the fungal-infected CNS *in vivo*, we generated mixed bone marrow chimeras in order to directly assess whether *Card9* ^*-/-*^ neutrophils have a cell-intrinsic chemotactic defect. WT animals were lethally irradiated and subsequently injected with a 1:1 ratio of CD45.1^+^ WT and CD45.2^+^ *Card9* ^*-/-*^ bone marrow cells. Following the adoptive transfer, animals were left to reconstitute for 8 weeks prior to *C*. *albicans* infection (S9 Fig). The relative recruitment of WT and *Card9* ^*-/-*^ neutrophils to the brain of infected chimeras was then assessed by FACS, with congenic CD45 isoform expression used to distinguish between WT and *Card9* ^*-/-*^ neutrophils. Using this approach, we found no defect in the ability of *Card9* ^*-/-*^ neutrophils to traffic into the infected brain, since the relative ratio of WT:*Card9* ^*-/-*^ neutrophils remained unchanged before and after infection (Fig 7A). These data indicate that neutrophil-intrinsic Card9 is dispensable for neutrophil recruitment during fungal infection of the CNS.

The mixed chimera experiment results (Fig 7A) implied that *Card9* ^*-/-*^ neutrophils are able to upregulate key adhesion molecules that allow their transmigration from the blood into the infected brain. To further test this, we next analyzed whether expression of various adhesion molecules that are required for neutrophil adhesion and rolling on the endothelial surface for transmigration into inflamed tissues was disrupted in the absence of Card9. Using qRT-PCR on whole brain homogenates, we found that all adhesion molecules analyzed were induced in the brain of WT mice following infection (Fig 7B). While we observed a delayed induction in the expression of α-integrin X and α-integrin L in infected *Card9* ^*-/-*^ brains, these differences were not evident at 72 hours post-infection. Since β-integrin 2 induction was greater in *Card9* ^*-/-*^ brains at 72 hours post-infection, we assessed whether its induction in the *Candida*-infected brain is fungal load-dependent by infecting WT mice with high and low brain fungal burdens. Indeed, induction of β-integrin 2, ICAM-1 and L-selectin was significantly greater in animals with higher brain fungal burden, whereas induction of α-integrin X, α-integrin L and P-selectin was not fungal load-dependent (S10 Fig). These data suggest that the higher levels of β-integrin 2 and the trend toward higher induction of ICAM-1 observed in *Card9* ^*-/-*^ brains (Fig 7B) may reflect greater tissue fungal proliferation in these mice. Collectively, we did not observe consistent or profound defects in the expression of neutrophil-targeted adhesion molecules in the infected *Card9* ^*-/-*^ brain (Fig 7B).

The mixed chimera experiment results (Fig 7A) also suggested that *Card9* ^*-/-*^ neutrophils do not have a survival defect *in vivo*. To directly rule out the possibility that reduced neutrophil numbers in the *Card9* ^*-/-*^ brain is caused by enhanced neutrophil apoptosis and/or death following their recruitment into the CNS, we analyzed the extent of apoptosis and death in neutrophils recruited to the brain at 24 hours post-infection using Annexin-V and 7-AAD staining. We found that the frequency of apoptotic and dead neutrophils was similar in WT and *Card9* ^*-/-*^ brains (Fig 7C), in line with our patient data that showed that CARD9 is not a survival factor in human neutrophils (Fig 3E).

Taken together, these data show that Card9 deficiency does not adversely affect cell-intrinsic neutrophil trafficking to the brain, expression of neutrophil-targeted adhesion molecules, or neutrophil survival during *C*. *albicans* infection of the CNS in mice.

### CARD9 Is Required for Neutrophil-Targeted Chemokine Production in the Infected CNS

**Fig 8:**
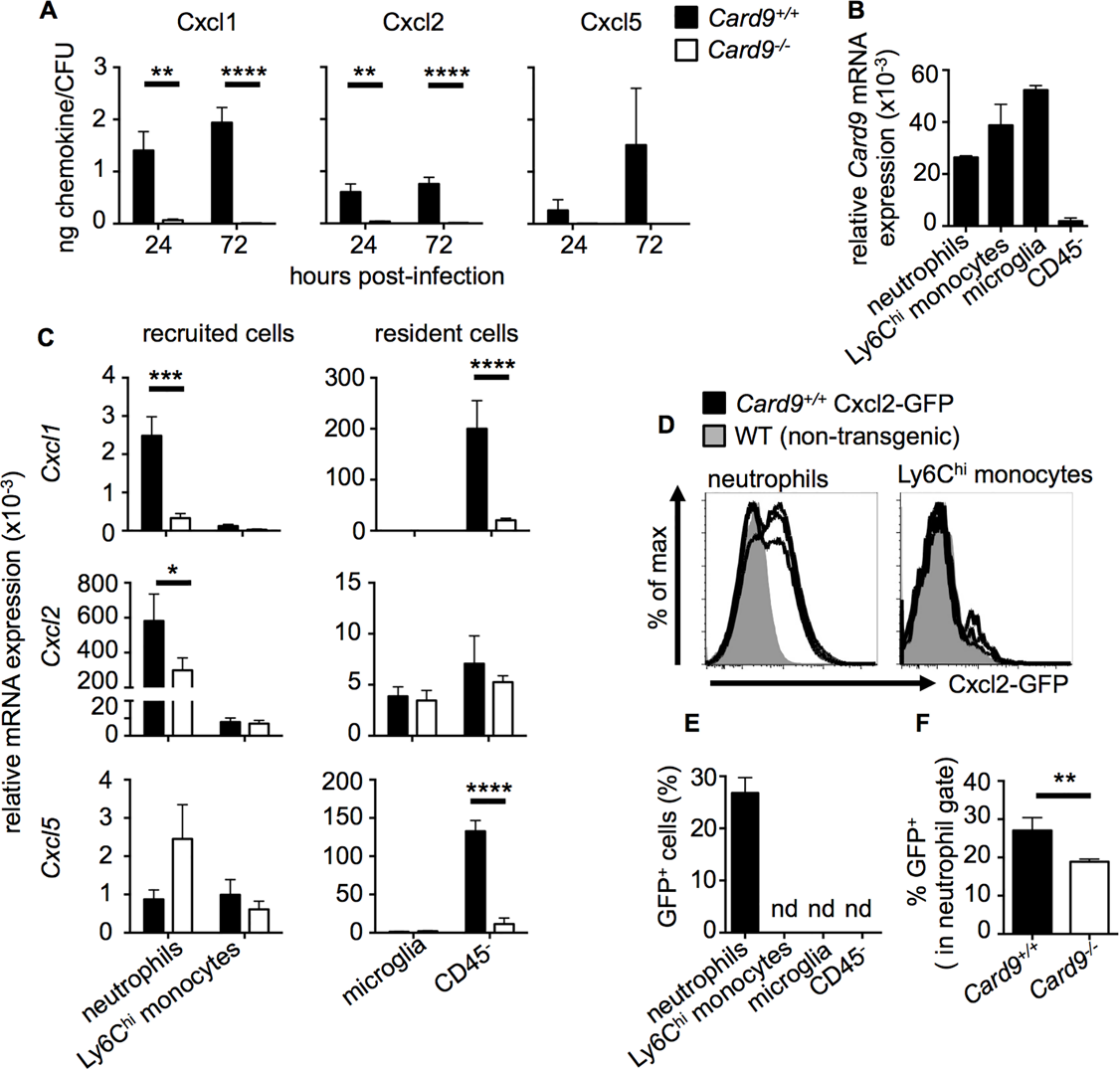
CARD9 is required for neutrophil-targeted chemokine production during fungal infection of the CNS. **(A**) Whole brain homogenates were analyzed for Cxcl1, Cxcl2 and Cxcl5 in WT (filled bars, n = 6) and *Card9* ^*-/-*^ (empty bars, n = 6) mice by Luminex array. Chemokine concentration (ng/g) was expressed relative to the mean brain fungal burden (CFU/g). Mice analyzed at 24 hours were infected with 1.3x10^5^ CFU SC5314, and mice analyzed at 72 hours infected with 7x10^4^ CFU. Data is pooled from two independent experiments and analyzed by two-way ANOVA (with Bonferroni correction). (**B**) Relative expression of *Card9* mRNA by indicated cell populations FACS-sorted from WT brains at 24 hours post-infection (n = 3). (**C**) Neutrophils, Ly6C^hi^ monocytes, microglia and CD45^-^ cells were FACS-sorted from WT and *Card9* ^*-/-*^ brains at 24 hours post-infection (inoculum: 1.3x10^5^) and RNA purified for qRT-PCR analysis of *Cxcl1*, *Cxcl2* and *Cxcl5* expression. See S11 Fig for purity analysis. Data is pooled from nine (neutrophils, Ly6C^hi^ monocytes, microglia) or six sorts (CD45^-^ cells) and analyzed by two-way ANOVA (with Bonferroni correction). (**D**) Transgenic Cxcl2-GFP reporter animals (black histograms) were infected with 1.3x10^5^ CFU and brain leukocytes analyzed by FACS at 24 hours post-infection. Expression of GFP within the indicated cell populations was compared to non-transgenic WT animals (grey histograms). Histograms are representative of two independent experiments. (**E**) Quantification of the frequency of GFP^+^ cells within the indicated cell populations and (**F**) percentage of GFP^+^ neutrophils within the WT and *Card9* ^*-/-*^ brain; nd: not detected. Data pooled from two independent experiments (n = 5) and analyzed by unpaired t-tests; data represent mean ± SEM. *P<0.05, **P<0.01, ***P<0.005, ****P<0.001.

Since *Card9* ^*-/-*^ neutrophils did not exhibit a cell-intrinsic chemotactic defect, and as we found our patient’s infected CSF to contain low concentrations of neutrophil-targeted CXC chemokines, we next investigated if this defect could also be modeled in the brains of *Card9* ^*-/-*^ mice. Thus, we analyzed whole brain homogenates for expression of Cxcl1, Cxcl2 and Cxcl5 by Luminex array in uninfected and *C*. *albicans*-infected mice. We found that both Cxcl1 and Cxcl2 were significantly induced following infection in both WT and *Card9* ^*-/-*^ mice whereas Cxcl5 was poorly induced (S11 Fig). At 24 hours post-infection, WT and *Card9* ^*-/-*^ brains had similar levels of these chemokines despite a 10-fold higher fungal load in *Card9* ^*-/-*^ brain tissue (S11 Fig). At 72 hours post-infection, chemokine levels decreased in WT mice in parallel with falling tissue fungal burden (Fig 6B), whereas chemokine levels in *Card9* ^*-/-*^ brains remained stable relative to 24 hours post-infection (S11 Fig) despite a further dramatic increase in brain fungal burden (Fig 6B). When chemokine production was expressed as a concentration relative to fungal load, Cxcl1 and Cxcl2 levels were dramatically decreased in *Card9* ^*-/-*^ brains (Fig 8A).

We then examined CXC chemokine induction in the kidney and found that Cxcl1 and Cxcl2 were significantly increased in the infected *Card9* ^*-/-*^ kidneys compared to WT (S6C Fig). Although induction of Cxcl1 and Cxcl2 was to a degree impaired relative to the kidney fungal load in *Card9* ^*-/-*^ mice (S6A and S6C Fig), renal CXC chemokine induction was sufficient for mediating neutrophil recruitment into the *Candida*-infected *Card9* ^*-/-*^ kidney (S6B Fig). Since the IL-17/IL-22 pathway has also been shown to promote neutrophil recruitment into infected tissues [34], and some CARD9-deficient patients have reduced Th17 cells in the peripheral blood [7, 9, 13], we determined the levels of IL-17 and IL-22 in the brains of WT and *Card9* ^*-/-*^ mice following infection. However, we found no consistent induction of these cytokines compared to uninfected control brain, at either the mRNA or protein levels. Taken together, *Card9* ^*-/-*^ mice display a marked insufficiency in the induction of Cxcl1 and Cxcl2 in the brain during *C*. *albicans* infection relative to the extent of tissue fungal burden.

### Neutrophils and CD45^-^ Cells Produce Neutrophil-Targeted Chemoattractants in a Card9-Dependent Manner

Since we identified that CXC chemokines were reduced in the CNS of both mice and humans with CARD9 deficiency, we next sought to understand the cellular source of these chemokines in a bid to further define the antifungal immune response within the brain. We used FACS-sorting (S12 Fig) to purify the major recruited myeloid cell populations from infected brains (neutrophils and Ly6C^hi^ monocytes), and then used qRT-PCR to quantify relative expression of *Cxcl1*, *Cxcl2* and *Cxcl5*. We also FACS-sorted and analyzed resident cell populations from infected brains, as microglia and astrocytes have been shown to be major producers of cytokines and chemokines during CNS inflammation [35–37]. Firstly, we found that all cell populations expressed *Card9*, with the highest levels detected in resident microglia (Fig 8B). Interestingly we also found low, albeit detectable, expression of *Card9* in the CD45^-^ population (Fig 8B), which is predominantly made up of resident glial cells as determined by the presence of transcripts for glial acidic fibrillary protein (*Gfap*; astrocyte marker) and myelin oligodendrocyte glycoprotein (*Mog*; oligodendrocyte marker), while endothelial cells were absent as determined by CD31/CD102 staining (S12C and S12D Fig). We found that neutrophils were the major cellular source of Cxcl2 in the infected brain in WT animals, while CD45^-^ cells predominantly produced Cxcl1 and Cxcl5 (Fig 8C). In infected *Card9* ^*-/-*^ brains, we found a significant decrease in levels of *Cxcl1* and *Cxcl2* from purified *Card9* ^*-/-*^ neutrophils, and further profound decreases in *Cxcl1* and *Cxcl5* in the *Card9* ^*-/-*^ CD45^-^ population (Fig 8C).

To visualize the cellular sources of Cxcl2 in the *Candida*-infected brain *in vivo*, we made use of a recently described transgenic Cxcl2-GFP reporter mouse [38]. In line with our qRT-PCR data, we found that neutrophils in the infected WT brain were the predominant producers of Cxcl2 following infection, whereas Cxcl2 was undetectable in other tested cell populations (Fig 8D and 8E). When we infected *Card9* ^*-/-*^ mice on this genetic background (*Card9* ^*-/-*^ Cxcl2-GFP), we found decreased proportion of Cxcl2-expressing *Card9* ^*-/-*^ neutrophils in the infected brain (Fig 8F). Thus, our data indicate that resident CD45^-^ cells and recruited neutrophils are the major producers of neutrophil-targeted CXC chemokines in the *C*. *albicans*-infected mouse brain. Taken together, because CNS tissue does not contain large numbers of resident neutrophils at steady state, our data are consistent with a model by which an early wave of Card9-dependent chemotactic signals by resident cells is followed by a subsequent positive feedback loop of neutrophil-driven cell chemoattraction that is defective in the *Card9* ^*-/-*^ brain.

## Discussion

In the present study, we show that the antifungal adaptor molecule CARD9 is critical for neutrophil recruitment from the blood into the CNS during fungal infection in mice and humans. CARD9 appears to act by promoting production of neutrophil-targeted chemoattractants in the infected CNS tissue, while cell-intrinsic neutrophil chemotaxis is CARD9-independent. Our conclusions are based on detailed analysis of differences in clinical, microbiological, pathological, immunological and molecular parameters between *Card9* ^*+/+*^ and *Card9* ^*-/-*^ mice, and are corroborated by analogous functional defects identified in a CARD9-deficient patient with *Candida* meningoencephalitis. Our study is the first to uncover the crucial role of CARD9 on neutrophil accumulation in the CNS during systemic fungal infection in mice and humans, and provides novel insight into the mechanisms of spontaneous susceptibility to CNS fungal infection seen in autosomal recessive CARD9 deficiency.

Here, we describe a novel *CARD9* missense mutation, c.170G>A (p.R57H), which further adds to the list of *CARD9* mutations associated with the development of systemic fungal infections [7–14, 18]. Multiple missense and nonsense *CARD9* mutations have now been described that reside in both the coiled-coil and CARD domains [7–14]. There has been thus far no correlation between specific *CARD9* mutations and development of systemic fungal infections, and therefore more research will be required to define the potential differential functional roles of the coiled-coil and CARD domains of CARD9 on promoting mucosal versus systemic immunity against *C*. *albicans* and other fungi.

Despite several reports of CARD9-deficient patients with CNS infections, the mechanisms of susceptibility in these patients are not well understood, which prompted us to investigate phagocyte function, since these cells are required components of systemic antifungal immune defense against *Candida* species [24]. We found that monocytes from the CARD9-deficient patient had a profound defect in pro-inflammatory cytokine production specifically in response to fungal components, similar to other reported findings [7–14]. In addition, we show for the first time a modest, yet significant, defect in killing of *C*. *albicans* yeasts by monocytes, the mechanisms of which merit future investigation. Similarly, in agreement with a previous report [7], we found a defect in killing of unopsonized yeasts by CARD9-deficient human neutrophils, although opsonization could restore this defect. We found no impact of CARD9 deficiency on the killing of hyphal *C*. *albicans* forms by either neutrophils or monocytes. These results confirm recently reported findings using CARD9-deficient neutrophils from a patient infected with *Phialophora*, in which only killing of unopsonized conidia was defective, while hyphal killing was intact [14]. Collectively, the impaired killing of CARD9-deficient neutrophils against unopsonized *Candida* yeasts has led to the hypothesis that CARD9 deficiency may predispose humans to CNS infection since opsonization is suboptimal in this tissue. However, because the predominant *Candida* morphology in the infected brain in mice and humans is hyphae (Fig 1A, Fig 6C and S4 Fig) [9, 24], and because phagocyte hyphal killing is intact in these patients, this current explanation appears insufficient to fully account for why these patients develop spontaneous CNS fungal disease.

Strikingly, we found that CARD9 deficiency resulted in a profound absence of neutrophils in the infected CNS (Fig 3, Fig 6 and S4 Fig); instead, eosinophils and lymphocytes were the predominant leukocytes in the infected CSF, in agreement with recently published findings in other CARD9-deficient patients with *Candida* CNS infection [7, 9]. This profound lack of neutrophil accumulation in the infected CNS is in stark contrast to the robust accumulation of neutrophils in the *Candida*-infected CNS of mice and humans without CARD9 deficiency [24, 29, 39]. In our CARD9-deficient patient, this CNS-specific neutropenia could not be explained by a decrease in circulating neutrophils or neutrophil survival, nor a cell-intrinsic chemotaxis defect. Instead, we found that neutrophil-targeted CXC chemokines were not induced in the infected CSF. In patients with neutrophilic meningitis, levels of IL-8 in the CSF have been reported to reach up to 45,000 pg/mL (median, 13,900 pg/mL) [30], and the infected CSF has been shown to be chemotactic toward neutrophils *ex vivo* [40]. Instead, our patient’s CSF lacked chemotactic activity toward neutrophils *ex vivo* and had approximately 300 pg/mL of IL-8, which is within the range of concentrations seen in the CSF of uninfected control individuals [30], and less than 20 pg/mL of CXCL1, CXCL2 and CXCL5. This is in contrast to the induction of CXC chemokines in the *Candida*-infected CSF of another patient without CARD9 deficiency who developed meningitis post-Ommaya reservoir placement at NIH, whose CSF was chemotactic toward neutrophils *ex vivo*. Therefore, our data indicate that CARD9 is required for proper production of these chemoattractants in response to CNS fungal infection. However, more work assessing CSF leukocyte immunophenotype and measuring these chemoattractants in the CSF of additional patients with CNS fungal infections, with and without CARD9-deficiency, is needed.

To directly examine the role of CARD9 in CNS antifungal defense, we moved to a mouse model of systemic candidiasis using *Card9* ^*-/-*^ mice [41]. We show for the first time that these animals develop significant brain fungal infection with a profound absence of neutrophil accumulation in the infected CNS. Furthermore, we also found that *Card9* ^*-/-*^ neutrophils could mobilize from the bone marrow into the blood, thus demonstrating a Card9-dependent defect of cell trafficking from the blood into the CNS, which is tissue- and fungus-specific. In line with our human data, we showed that *Card9* ^*-/-*^ neutrophils did not have a survival defect nor were they intrinsically defective in trafficking from the blood into the *Candida*-infected brain, since they were able to mobilize into WT brains in mixed bone marrow chimera experiments.

Since our human data suggested that neutrophil-targeted chemokine production depended on CARD9, we investigated the production and source of these molecules in the infected CNS using our mouse model. We found that production of neutrophil-targeted chemokines was grossly suboptimal in the *Card9* ^*-/-*^ brain relative to the tissue fungal burden. These data suggest that the lack of neutrophils in the Card9-deficient CNS is caused, at least in part, by impaired chemoattractant production. Recent work has demonstrated dependency on both Card9 and IL-1R/Myd88 signaling for neutrophil-targeted chemokine production and neutrophil accumulation in the *Aspergillus*-infected mouse lung [38, 42]. Specifically, *Card9* ^*-/-*^ mice infected with *Aspergillus* recruited normal numbers of neutrophils to the lung in the early phase of the infection driven via IL-1R/Myd88–dependent CXC chemokine production, whereas at the later stages of the infection a 40% decrease in neutrophil accumulation was observed, which was due to decreased CXC chemokine production by *Card9* ^*-/-*^ hematopoietic cells [38, 42]. In stark contrast to this partial dependency on Card9 for neutrophil recruitment during pulmonary aspergillosis, we show here that Card9 deficiency almost abolishes neutrophil recruitment to the CNS throughout the entire course of *C*. *albicans* infection. Taken together, these data further underscore the tissue-specific nature of antifungal innate immune responses [24] and provide a potential explanation why CARD9-deficient patients, including our patient reported here, have not been reported to have increased susceptibility to pulmonary infections with ubiquitous inhaled molds.

We next investigated the cellular source of neutrophil-targeted chemokines in the CNS, in a bid to further understand how neutrophils are recruited to this site during fungal infection. We found that neutrophils were the predominant source of Cxcl2 and to a lesser extent of Cxcl1, in agreement with previous reports that neutrophils can create positive-feedback loops for their continued recruitment into fungal-infected tissue [32]. We next investigated if Card9 was required for this phenomenon, and found that *Card9* ^*-/-*^ neutrophils produced less Cxcl1 and Cxcl2. Interestingly, we also found significant decreases in Cxcl1 and Cxcl5 transcript levels in the CD45^-^ resident cell population purified from *Card9* ^*-/-*^ brains, which we found were positive for transcripts encoding glial markers *Gfap* and *Mog*. Therefore, our data point to a two-step chemokine-driven neutrophil recruitment process in the WT CNS, initially promoted by resident stromal cells and followed by cell-intrinsic neutrophil chemokine production. In *Card9* ^*-/-*^ brains, an initial ‘hit’ of defective chemokine production by the resident CD45^-^ compartment may lead to poor neutrophil recruitment, which appears to be further exacerbated by the absence of neutrophil-mediated Cxcl1 and Cxcl2 production in the *Card9* ^*-/-*^ brain.

Future studies should also aim to examine other neutrophil-targeted chemoattractants that may also contribute to Card9-dependent neutrophil recruitment into the infected CNS, as well as their myeloid and non-myeloid cellular sources and their Card9-dependent organ-specific localization in tissue. In particular, our work has indicated that the resident cells in the brain are integral for neutrophil-targeted chemokine production, and their identification and Card9-dependent functions within antifungal immunity is an area of ongoing investigation in our laboratory. Additionally, future work should also focus on whether CARD9 is equally critical for neutrophil recruitment to the CNS in CARD9-deficient patients and mice infected with other fungal pathogens, such as the recently reported *Exophiala* [10].

In summary, we have identified a novel critical role for CARD9 in tissue- and fungus-specific neutrophil recruitment to the CNS during *C*. *albicans* infection in mice and humans. CARD9 is important for the production of chemoattractants in the CNS by neutrophils and resident cells, whereas other neutrophil-intrinsic functions such as phagocytosis, oxidative burst, killing, chemotaxis and survival, appear largely CARD9-independent. Our work highlights the importance of the CLR/CARD9 immune pathway in the neutrophil-dependent control of *C*. *albicans* infection of the CNS, and forms the foundation for devising immune-based therapies for bypassing CARD9 in the production of neutrophil-targeted chemokines such as via the potential direct intrathecal delivery of these molecules in infected CARD9-deficient patients.

## Methods

### Ethics

The patient, her relatives and the healthy donors were enrolled in protocols approved by the National Institute of Allergy and Infectious Diseases and National Cancer Institute Institutional Board Review (IRB) committees, and provided written informed consent for participation in the study. This study was conducted in accordance with the Helsinki Declaration. Animal studies were performed in accordance with the recommendations in the Guide for the Care and Use of Laboratory Animals of the National Institutes of Health, under the auspices of protocol LCID14E approved by the Animal Care and Use Committee of the National Institute of Allergy and Infectious Diseases. Animals were euthanized by cervical dislocation following administration of ketamine/xylazine cocktail.

### Isolation of DNA and *CARD9* Sequencing

DNA was harvested from Ammonium-Chloride-Potassium (ACK)-lysed whole blood from the CARD9-deficient patient, her parents and two siblings using the Gentra Puregene Blood DNA isolation Kit (Qiagen) per the manufacturer’s instructions. Genomic amplification was performed in 15 μL reactions using Platinum PCR SuperMix High Fidelity (Life Technologies), 0.625 mM of each primer and 10–200 ng of DNA. Cycling conditions were 95°C for 3 minutes followed by 35 cycles of 95°C for 20 seconds, and 68°C for 2:45. PCR products were purified using ExoSAP-IT (USB products by Affymetrix), sequenced using Big Dye Terminators v3.1 (Life Technologies), cleaned up using Performa DTR Ultra spin plates and run on an ABI 3730XL. Amplification and sequencing primers are shown in S1 Table.

### Isolation of Human PBMC, CD14^+^ Monocytes and Neutrophils from Peripheral Whole Blood

PBMC were harvested from whole blood by gradient centrifugation using Lymphocyte Separation Media (Lonza), according to the manufacturer’s instructions. PBMC were washed in PBS and quantified prior to use in stimulation assays and flow cytometry (see below) or downstream sorting of CD14^+^ monocytes. CD14^+^ monocytes were magnetically sorted using negative selection via the Monocyte Isolation Kit II (Miltenyi) according to the manufacturer’s instructions. Purity and viability of sorted monocytes was >84% and >92%, respectively. Neutrophils were isolated using 3% Dextran in 0.85% sodium chloride and red blood cells lysed using sequential exposure to 0.2% and 1.6% NaCl solutions. Purity and viability of isolated neutrophils exceeded 95%. CD14^+^ monocytes and neutrophils were quantified prior to use in FACS or Western blot analyses or downstream functional assays, including internalization assays, killing assays, survival studies and chemotaxis studies (see below).

### Quantification of Immune Cells in the Infected CSF by FACS Analysis

CSF was centrifuged and cells were washed, then stained with a Live/Dead fluorescent dye (Invitrogen) for 10 minutes in PBS at 4°C, followed by Fc receptor blockade with Fc Blocking Reagent (Miltenyi) in FACS buffer (PBS supplemented with 0.5% BSA and 0.01% sodium azide) at 4°C. Surface antigen staining was then performed by incubating cells with fluorochrome-conjugated (eFluor 605 NC, PE-Cy7, APC, APC-eFluor 780, Alexa Fluor 700, eFluor 450, FITC, PE, PerCP-Cy5.5, Biotin/Streptavidin Brilliant Violet 570) antibodies against human CD45 (HI30), CD56 (CMSSB), CD3 (SK7), CD19 (HIB19), CD11b (ICRF44), CD11c (3.9), CD123 (6H6) (eBioscience); CD14 (M5E2), CD16 (3G8), HLA-DR (G46-6) (BD Biosciences); CD141 (M80) (BioLegend) for 30 minutes on ice. After incubation, the cells were washed 3 times with FACS buffer and sample acquired using a 5-laser BD LSRFortessa, equipped with BD FACS Diva software (BD Biosciences). FlowJo (TreeStar) was used for the final analysis. Cell numbers were quantified using PE-conjugated fluorescent counting beads (Sperotech).

### PBMC Stimulation and Cytokine Determination by Luminex Array

To determine whether CARD9 deficiency affects the ability of PBMCs to produce pro-inflammatory cytokines and chemokines, we used a Luminex-based assay. In brief, 5 x 10^5^ PBMCs from healthy donors or the CARD9-deficient patient were incubated in duplicate in a round-bottom 96-well plate (Corning Inc.) at 37°C in a 5% CO_2_ incubator in RPMI 1640 containing 10% fetal bovine serum (FBS, Gibco), 100 U/mL of penicillin, and 100 μg/mL of streptomycin (unstimulated), or RPMI 1640 with 10% FBS/antibiotics containing LPS (100 ng/mL), or heat-killed *C*. *albicans* SC5314 yeasts (1 x 10^6^ /mL). After 48 hours of stimulation, PBMCs were pelleted and the supernatant was collected and stored at -80°C until analysis. Luminex analysis was done via a multiplex bead array assay with antibodies and cytokine standards to generate known concentration curves (R&D Systems, Peprotech). Individual Luminex bead sets (Luminex) were coupled to cytokine-specific capture antibodies according to manufacturer’s protocols and biotinylated polyclonal antibodies were used at twice the recommended concentrations for a classical ELISA according to the manufacturer’s instructions. The assay was run with 1200 beads per set of cytokines in a volume of 50 μL. The plates were read on a Luminex MAGPIX platform where more than 50 beads were collected per bead set. The median fluorescence intensity of the beads was then measured for each individual bead, which was analyzed with the Millipex software using a 5P regression algorithm.

### *Candida* Internalization by Human Neutrophils and CD14^+^ Monocytes

To determine whether CARD9 deficiency affects the capacity of peripheral blood neutrophils and/or CD14^+^ monocytes to internalize *C*. *albicans*, 5 x 10^4^ cells from healthy donors or the CARD9-deficient patient were incubated in a polystyrene round-bottom FACS tube (BD Falcon) in a 37°C shaking water bath for 60 minutes in RPMI 1640 containing 100 U/mL of penicillin and 100 μg/mL of streptomycin with dTomato-expressing *Candida* that had been suspended in RPMI 1640 with antibiotics without (unopsonized) or with 5% human serum (opsonized) at a cell:yeast ratio of 1:25 or 1:1, respectively. Samples were then stained with a FITC-conjugated anti-*Candida* antibody (cat #21164; Abcam) and APC-conjugated anti-CD14 (for monocytes) or anti-CD15 (for neutrophils) antibodies and subsequently washed. FACS was performed on a 5-laser LSRFortessa. The percentage of *Candida* internalization by myeloid cells was then determined by initially gating on APC^+^ monocytes or neutrophils and then by defining the percentage of APC^+^ cells that had internalized *Candida* (APC^+^PE^+^FITC^-^) versus those that had *Candida* bound on the cell surface (APC^+^PE^+^FITC^+^).

### *Candida* Killing by Human Neutrophils and CD14^+^ Monocytes

To determine whether CARD9 deficiency affects the capacity of peripheral blood neutrophils and/or CD14^+^ monocytes to kill *Candida*, we used the alamarBlue-based fluorescence assay as previously described [22]. Briefly, neutrophils and monocytes were harvested from peripheral blood as described above, and 5 x 10^4^ cells from healthy donors or the CARD9-deficient patient were incubated in flat-bottom 96-well plates (Genesee Scientific) for 2.5 hours with opsonized or unopsonized *Candida* yeasts or 3-hour pre-grown pseudohyphae at a cell:*Candida* ratio ranging from 1:4 to 4:1 (see figure legends). The wells were then treated with 0.02% Triton X-100 in water to lyse the neutrophils/monocytes, washed twice with PBS and incubated with 1x alamarBlue (Invitrogen) in PBS for 18 hours at 37°C. Fluorescence was measured using a POLARstar OPTIMA plate reader (BMG Labtech). Killing was calculated by comparing the fluorescence of *Candida* incubated with neutrophils or monocytes with that of *Candida* incubated without myeloid cells.

### Survival Studies of Human Neutrophils

Neutrophils from healthy donors and the CARD9-deficient patient were harvested as described above. To determine whether CARD9 deficiency impairs survival of neutrophils, cells were cultured for 3 or 6 hours at 37°C in a 5% CO_2_ incubator in RPMI 1640 without FBS (serum starvation) or in RPMI 1640 with 10% FBS with tumor necrosis factor-α (50 ng/mL) or live *Candida albicans* SC5314 (1 x 10^5^ cells/mL) or in infected CSF from the CARD9-deficient patient to assess the extent of cell apoptosis and death. Cells were stained with Annexin V and propidium iodide (BD Biosciences) per the manufacturer’s instructions, and flow cytometry was performed on a 5-laser LSRFortessa.

### Chemotaxis Assays of Neutrophils

Chemotaxis assays were performed on freshly isolated neutrophils using the EZ-TAXIScan (ECI, Inc., Japan) or a modified Boyden chamber (NeuroProbe). In brief, 5 x 10^3^ neutrophils were placed in each well of the EZ-TAXIScan and 1.0 μL of either buffer or fMLF (5x10^-8^ M) or IL-8 (5x10^-8^ M) was added to the opposing well. Images of cellular migration were captured every 30 sec for 60 min at 37°C and individual cells (10 picked at random) were tracked digitally using ImageJ software. The paths of the migrating cells were plotted with the position of each cell at t = 0 anchored at the origin. Using the coordinates obtained at each time point, the migratory path of each cell was resolved into a random migrational vector (orthogonal to the direction of the chemoattractant, along the x-axis) and a directed migrational vector (parallel to the direction of the chemoattractant, along the y-axis). In the absence of chemoattractant, the directed migrational vector should be equivalent to the random migrational vector. For studies analyzing chemotaxis to several chemoattractants simultaneously, chemoattractants (fMLF, LTB_4_, C5a, and IL-8) were prepared at the indicated concentrations in PBS with 0.1% human serum albumin and loaded in triplicate to the bottom well of a 96-well ChemoTx disposable chemotaxis plate (Neuro Probe, Inc.). Neutrophils were labeled with calcein-AM (10 μg/mL) and 7.5 x10^4^ added on top of the wells, separated from the chemoattractants by a 5 μm polycarbonate filter. Plates were incubated for 45 min at 37°C, and the fluid in upper chamber removed. Cells attached to the underside of the filter were detached by washing in 2 mM EDTA. After removing the filter, fluorescence of each well was determined in a multi-well fluorescent plate reader (SpectraMAX Gemini EM). The number of migrating cells was determined using a standard curve made with known quantities of labeled cells. To test whether CSF from our CARD9-deficient *Candida*-infected patient or CSF from a healthy uninfected donor or CSF from our CARD9^+/+^ *Candida*-infected patient post-Ommaya reservoir placement or CSF from two patients with non-neutrophilic (lymphocytic) meningitis was chemotactic itself toward WT and/or CARD9-deficient neutrophils, the NeuroProbe protocol was used, using a 50% dilution of CSF as the chemoattractant.

### Expression of CARD9 on Neutrophils and CD14^+^ Monocytes Using FACS Analysis

Neutrophils and PBMCs were harvested from healthy donors and the CARD9-deficient patient as described above. Cells were first stained with Live/Dead fluorescent dye (Invitrogen) for 10 minutes on ice, followed by anti-CD16/32 for 10 min to block Fc receptors, and then by APC-conjugated anti-human CD14 (clone M5E2; BD Biosciences). Cells were then fixed with 2% paraformaldehyde (Affymetrix) at room temperature for 10 minutes and subsequently permeabilized with 1X FACS permeabilizing solution 2 (BD Biosciences) according to the manufacturer’s instructions. The cells were then incubated for 30 minutes with rabbit monoclonal anti-CARD9 (clone EPR6489; Origene) or monoclonal rabbit IgG isotype control (clone EPR25A; Abcam), washed and then stained with PE-conjugated secondary anti-rabbit antibody (eBioscience) for 30 minutes. Samples were acquired using a BD LSRFortessa.

### Expression of CARD9 on Neutrophils and CD14^+^ Monocytes Using Western Blot Analysis

Neutrophils and CD14^+^ monocytes were harvested from healthy donors and the CARD9-deficient patient as described above. Cells were washed once with ice-cold PBS and lysed in buffer containing 50 mM HEPES, 50 mM NaCl, 10% glycerol, 0.5% Nonidet P-40, 2 mM EDTA, and a Protease and Phosphatase Inhibitor Cocktail (cat# 78440; Thermo Scientific). Cell lysates were centrifuged at 13,200 g for 10 minutes at 4°C, and equal protein amounts from the supernatant were resuspended in SDS loading buffer with 5% β-mercaptoethanol for Western blot analysis. Proteins were analyzed by SDS-PAGE and transferred to Immobilon P, polyvinylidene difluoride membrane (Millipore, Billerica). Membranes were blocked in TBS-T containing 5% nonfat dry milk, and then incubated with polyclonal rabbit anti-human CARD9 (cat# 12892-1-AP; ProteinTech Group, Chicago, IL) and rabbit monoclonal antibody against GAPDH (cat# 2118; Cell Signaling Technology) in TBS-T containing 5% BSA for 2 hours at room temperature under continuous agitation. Membranes were then washed 3 times with TBS-T, and incubated with horseradish peroxidase–conjugated anti-rabbit secondary antibody (Southern Biotech) in TBS-T containing 5% nonfat dry milk for 1 hour at room temperature. Membranes were subsequently washed, and detection of immunoreactive bands was performed using the SuperSignal West Fempto Maximum Sensitivity Substrate chemiluminescence (ECL) detection kit according to the manufacturer’s instructions (Pierce Biotechnology).

### Determination of IL-17A^+^ CD4^+^ T Cells

PBMCs from healthy donors and the CARD9-deficient patient were isolated as described above and cultured in RPMI 1640 with 10% FBS, 2mM L-glutamine, 100 U/mL of penicillin, and 100 μg/mL of streptomycin (Gibco) at 37°C in a humidified 5% CO_2_ incubator. Intracellular staining for IL-17A was performed on PBMCs stimulated for 6 hours with PMA (20 ng/ml; Sigma) and ionomycin (1 μM; Life Technologies) in the presence of Brefeldin A (10 μg/ml; Sigma). FITC-labeled anti-CD4 (clone RPA-T4; eBioscience) was added for the final 30 minutes of culture before the fix and permeabilization step. Cells were then washed with PBS and then fixed and made permeable with Foxp3 staining set (eBioscience) according to the manufacturer's instructions. Cells were then incubated with PE-labeled anti-IL-17A (clone eBio64CAP17; eBioscience) for 1 hour at 4°C, and acquired on a FACSCanto (BD Biosciences).

### Mice and Systemic Candidiasis Model

8–12 week old female C57BL/6 (Taconic) and *Card9* ^*-/-*^ [41] mice were maintained in individually ventilated cages under specific pathogen-free conditions at the National Institutes of Health (Bethesda, MD, USA). Cxcl2-GFP reporter animals [38] were bred and housed in the Memorial Sloan Kettering Cancer Center Comparative Medicine Shared Resources under specific pathogen-free conditions. *Candida albicans* strain SC5314 was used for all infections. Yeast was serially passaged 3 times in YPD (yeast extract, bacto-peptone and dextrose) broth, grown at 30°C with shaking for 18–24 hours at each passage. Yeast cells were washed in PBS, counted, and injected intravenously via the lateral tail vein. Each animal received 0.7–1.3 x 10^5^ yeast cells (details for doses used in individual experiments are presented in the figure legends). At 24 or 72 hours post-infection, animals were euthanized and following analyses performed: brain fungal burdens; FACS analysis on blood, brain and bone marrow cells; quantification of chemokines by Luminex array; histopathology; cell sorting; RNA isolation from brain tissue followed by qRT-PCR analysis.

### Depletion of Neutrophils in Infected Mice by 1A8 Administration

WT mice were given 100 μg of 1A8 or 2A3 (isotype) antibody (Bio X Cell) intravenously at 24 hours prior to infection, and again at the time of infection. Antibody-treated mice were infected with 1 x 10^5^ CFU of C. albicans SC5314, and analyzed at 24 hours post-infection for brain fungal burden and FACS analysis (see below).

### Generation of Mixed Bone Marrow Chimeras

6–8 week old recipient C57BL/6.SJL (CD45.1^+^) mice were irradiated with 900 rad and left to rest for 4 hours. Bone marrow from gender-matched C57BL/6.SJL and *Card9* ^*-/-*^ (CD45.2^+^) donor mice was isolated from the femurs, washed and counted using Trypan blue exclusion, and resuspended as a 1:1 ratio for transfer. Flow cytometry was used to confirm the ratio of CD45.1^+^ to CD45.2^+^ cells prior to use. 5 x 10^6^ total bone marrow cells were transferred to each irradiated recipient mouse intravenously via the lateral tail vein approximately 4 hours post-irradiation. Recipient animals were given trimethoprim/sulfamethoxazole in the drinking water for the first 4 weeks of reconstitution, and then switched to antibiotic-free water. Mice were left to reconstitute for a total of 8 weeks post-transfer, and chimera status was assessed by flow cytometry on a sample of peripheral blood. Chimeras were then infected with 1.3 x 10^5^ CFU of SC5314, and euthanized for analysis at 24 or 72 hours post-infection. Brains and 300 μL peripheral blood were stained for flow cytometry analysis as above. WT and *Card9* ^*-/-*^ neutrophils (CD45^+^ CD11b^+^ Ly6G^hi^) were separated based on expression of congenic CD45.1 and CD45.2 markers and the ratio between them calculated.

### Determination of Fungal Burdens from Mouse Brains

At 24 or 72 hours post-infection, animals were euthanized and brains and kidneys were weighed, homogenized in PBS, and serially diluted before plating onto YPD agar supplemented with Penicillin/Streptomycin (Invitrogen). Colonies were counted after incubation at 37°C for 24–48 hours.

### Flow Cytometry of Mouse Bone Marrow, Blood and Brain

Leukocytes were isolated from brain, kidney, blood and bone marrow using previously described methods [24, 43], resuspended in PBS and stained with fluorochrome-conjugated antibodies as described for the human samples. Anti-mouse antibodies used in this study were: CD45 (30-F11), CD11b (M1/70), CD11c (N418), MHC Class II (M5/114.15.2), CD45.1 (A20), CD3 (145-2C11), CD19 (eBio1D3), F480 (BM8), all from eBiosciences, and CD45.2 (104), Ly6G (1A8), Ly6C (AL-21), all from BD Biosciences.

### FACS Analysis of Neutrophils for Annexin V and 7-AAD Expression

Brain-isolated leukocytes were Fc-blocked and stained with fluorochrome-conjugated antibodies as described above, washed twice with PBS, and resuspended in Annexin V binding buffer (BD Biosciences). Samples were incubated at room temperature for 15 minutes with 5 μl each of FITC-conjugated Annexin V and 7-AAD per the manufacturer’s instructions (BD Biosciences). FACS was performed on a 5-laser LSRFortessa.

### Chemokine/Cytokine Quantification in Mouse Brains by Luminex Array

Brains and kidneys were weighed and then homogenized in PBS/0.05% Tween20 and a protease inhibitor cocktail (Roche). Cell debris was removed by two sequential centrifugation steps (3600 rpm for 5 minutes, 13200 rpm for 10 minutes), and the resulting supernatant filtered through 0.2 μm filters, snap-frozen on dry ice and stored at -80°C until analysis. Samples were analyzed by Luminex assay, as described above, and cytokine/chemokine concentrations were determined per gram of tissue.

### Histopathology

Brains were removed from infected mice at the indicated time points and fixed in 10% formalin for 24 hours before embedding in paraffin wax. Tissue sections were stained with periodic acid-Schiff (PAS) and hematoxylin and eosin (H&E).

### FACS Sorting of Brain Myeloid and Non-myeloid Cells

WT animals were infected with 1.3 x 10^5^ CFU SC5314 and euthanized at 24 hours post-infection. Brains were isolated and leukocytes stained as above with sterile antibodies. Neutrophils (CD45^hi^ CD11b^+^ Ly6G^hi^ Ly6C^int^), Ly6C^hi^ monocytes (CD45^hi^ CD11b^+^ Ly6C^hi^ Ly6G^-^), microglia (CD45^lo^ CD11b^+^ Ly6G^-^ Ly6C^-^) and stromal cells (CD45^-^) were FACS-sorted into sterile sorting buffer (HBSS supplemented with 2 mM EDTA, 10% FCS, 100 U/mL penicillin, 100 μg/mL streptomycin) using a FACS Aria instrument. Purity of cells, on average, were as follows: neutrophils, 90%; monocytes, 88%; microglia, 95%; stromal cells, >99% (S12 Fig). Cells were then centrifuged (1500 rpm 5 minutes, 4°C) and resuspended in Trizol for RNA purification. 3–5 animals were pooled for individual sorts, and 3–6 independent sorts were performed in total.

### Analysis of Cxcl2 Expression by Brain Myeloid Cells Using Cxcl2-GFP Reporter Mice

Cxcl2-GFP reporter and non-transgenic WT littermates were infected as described above with 1.3 x 10^5^ CFU SC5314 intravenously and analyzed at 24 hours post-infection. Brains from infected mice were processed as previously described [24], and stained for FACS analysis as described above. Cxcl2-GFP expression was determined in the major myeloid populations (neutrophils, Ly6C^hi^ monocytes and microglia) by comparison with WT (GFP^-^) cells gated in the same way.

### Generation of cDNA and qRT-PCR

RNA was extracted from either sorted brain myeloid cells or homogenized brain tissue using Trizol (Invitrogen) and the RNeasy kit (Qiagen) per the manufacturer’s protocol. Purified RNA was used as a template for cDNA generation using the qScript cDNA SuperMix kit (Quanta Biosciences) with oligodT and random primers. Quantitative PCR was performed by SYBR Green (PerfeCTa SYBR Green FastMix ROX; Quanta BioSciences) or TaqMan detection (PerfeCTa qPCR FastMix ROX; Quanta BioSciences) with the 7900HT Fast Real-Time PCR System (Applied Biosystems). All qPCR assays were performed in duplicate and the relative gene expression of each gene was determined after normalization with GAPDH transcript levels using the ΔΔCT method. TaqMan primers/probes (*Sell*, *Selp*, *Icam1*, *Itgax*, *Itgb2*, *Itgal*, *Gfap*, *Mog*, *Card9*, *Il17a*, *Il22*, *Gapdh*) were predesigned by Applied Biosystems. Primers for SYBR Green detection were as follows: *Gapdh*: FOR 5’-aactttggcattgtggaagg, REV 5’-acacattgggggtaggaaca; *Cxcl1*: FOR 5’-actgggattcacctcaagaa, REV 5’-tctccgttacttggggacac; *Cxcl2*: FOR 5’-aagtttgccttgaccctgaa, REV 5’-aggcacatcaggtacgatcc; *Cxcl5*: FOR 5’-gaaagctaagcggaatgcac, REV 5’-gggacaatggtttccctttt.

### *Staphylococcus aureus* Infection Model

Community-acquired methicillin resistant *Staphylococcus aureus* USA300 (CA-MRSA, LAC strain from F. DeLeo, Rocky Mountain Laboratories, NIAID) was grown in brain heart infusion broth (BHI, Difco Laboratories, Detroit, Mich.) at 37°C with shaking at 230 rpm for 18 hours. The culture was then centrifuged at 3000 rpm for 10 minutes at room temperature and the pellet washed twice with PBS. WT and *Card9* ^*-/-*^ mice were infected intravenously with 2 x 10^7^ CFU via the lateral tail vein, and animals euthanized at 48 hours post-infection and analyzed as described above.

### Statistics

Statistical analyses were performed using GraphPad Prism 6.0 software. Details of individual tests are included in the figure legends. In general, data was tested for normal distribution by Kolmogorov-Smirnov normality test and analyzed accordingly by unpaired two-tailed t-test or Mann Whitney U-test. In cases where multiple data sets were analyzed, two-way ANOVA was used with Bonferroni correction. In all cases, *P* values <0.05 were considered significant.

## Supporting Information

S1 Fig Schematic diagram of the human *CARD9* gene domains and the *CARD9* mutations that have been reported to result in fungal disease. The CARD and coiled-coil (CC) domains are indicated along with reported *CARD9* mutations. The R57H mutation of our patient is indicated in red and the previously reported mutations associated with fungal infection of the CNS are indicated in blue.
(TIFF)

S2 Fig CARD9 is not expressed by CD4^+^ T-cells. FACS histogram of CARD9 staining in CD4^+^ T-cells from a healthy donor, compared to the isotype control.
(TIFF)

S3 Fig The c.170G>A *CARD9* missense mutation results in impaired production of pro-inflammatory mediators by PBMCs upon fungal stimulation. Cytokine (left panel) and chemokine (right panel) production by healthy donor (n = 10) and patient (n = 4) PBMCs after 48 hours of stimulation with heat-killed *C*. *albicans* (n = 4–10 independent experiments). Data is analyzed by Mann Whitney U-test or unpaired t-test, where appropriate. *P < 0.05; **P < 0.01; ***P < 0.001; ****P < 0.0001. Data represent mean ± SEM.
(TIFF)

S4 Fig CARD9 deficiency results in *Candida* fungal invasion in the CNS without neutrophil tissue infiltration. **(A)** Hematoxylin and eosin (H&E), **(B)** Grocott-Gomori methenamine-silver (GMS) and **(C)** Periodic acid-Schiff (PAS) stains of the patient’s brain biopsy sample showing filamentous fungal elements consistent with *C*. *albicans* and absence of neutrophil infiltration in the infected tissue (magnification, A, ×100; B-C, ×200).
(TIFF)

S5 Fig *Card9* ^*-/-*^ mice have reduced neutrophil frequency in the brain during fungal infection. (**A**) Neutrophil frequency was determined by FACS in the brain of WT (filled bars, 0 hours n = 5, 24 hours n = 6–13, 72 hours n = 6–13) and *Card9* ^-/-^ (empty bars, 0 hours n = 5–6, 24 hours n = 6–14, 72 hours n = 6–12) mice; data analyzed by two-way ANOVA. Example plots are from 24 hours post-infection and gated on live CD45^+^ single cells. (**B**) Relative frequencies of different subsets of hematopoietic CD45^+^ cells in the brain of WT (n = 6) and *Card9* ^*-/-*^ (n = 6) mice at 24 hours post-infection (dose: 1.3x10^5^ CFU) was determined by FACS. ‘Other (myeloid)’ cells represent eosinophils, dendritic cells and macrophages. Data is pooled from two independent experiments. **P<0.01, ***P<0.005 by two-way ANOVA. Data represent mean ± SEM.
(TIFF)

S6 Fig *Card9* ^*-/-*^ mice mobilize neutrophils into the *C*. *albicans* infected kidney. (**A**) Kidney fungal burdens in WT (filled circles) and *Card9* ^*-/-*^ (empty circles) mice at 24 (WT n = 12, KO n = 11) and 72 (WT n = 12, KO n = 10) hours post-infection; data pooled from 3–4 independent experiments and analyzed by Mann Whitney U-test. (**B**) Neutrophil numbers were determined by FACS in the left kidney of WT (filled bars, n = 6 per time point) and *Card9* ^-/-^ (empty bars, n = 6 per time point) mice; data analyzed by two-way ANOVA. (**C**) Whole kidney homogenates were analyzed for Cxcl1, Cxcl2 and Cxcl5 in uninfected and infected WT (filled bars, n = 6 per time point) and *Card9* ^*-/-*^ (empty bars, n = 6 per time point) mice by Luminex array. Data is pooled from two independent experiments and analyzed by two-way ANOVA. *P<0.05, **P<0.01, ***P<0.005, ****P<0.001. Data represent mean ± SEM.
(TIFF)

S7 Fig Card9 is dispensable for control of bacterial growth and neutrophil accumulation in the brain following *S*. *aureus* infection. WT (n = 7) and *Card9* ^*-/-*^ (n = 9) mice were infected intravenously with 2x10^7^ CFU of *S*. *aureus* and euthanized at 48 hours post-infection for (**A**) bacterial brain burden and (**B**) accumulation of neutrophils in the brain by FACS. Data is pooled from two independent experiments.
(TIFF)

S8 Fig WT and *Card9* ^*-/-*^ mice produce comparable numbers of neutrophils in the bone marrow, during steady state and post-infection. (**A**) The number of early neutrophil progenitors (Ly6G^int^ CD11b^+^) and (**B**) total neutrophils (Ly6G^int^ CD11b^+^ and Ly6G^hi^ CD11b^+^ combined) were determined by FACS in the bone marrow of WT (filled bars, 0 hours n = 6, 24 hours n = 6, 72 hours n = 7) and *Card9* ^-/-^ (empty bars, 0 hours n = 7, 24 hours n = 6, 72 hours n = 6) mice at indicated time points. Mice analyzed at 24 hours were infected with 1.3x10^5^ CFU SC5314, and mice analyzed at 72 hours infected with 7x10^4^ CFU. Data is pooled from 2–4 independent experiments and analyzed by two-way ANOVA. *P<0.05, **P<0.01. Data represent mean ± SEM.
(TIFF)

S9 Fig Schematic of mixed bone marrow chimera generation. WT CD45.1^+^ and *Card9* ^*-/-*^ CD45.2^+^ animals were used as donors for the isolation of bone marrow cells that were mixed in a 1:1 ratio prior to transfer. Mixed bone marrow was injected intravenously into irradiated WT CD45.1^+^ recipient mice, which were left to reconstitute for 8 weeks post-transfer. Chimera status was confirmed by FACS on a sample of peripheral blood prior to infection.
(TIFF)

S10 Fig Induction of neutrophil-targeted adhesion molecules in the *C*. *albicans* infected WT mouse brain. WT animals were systemically infected with a low (7x10^4^) or a high inoculum (1x10^6^) of *C*. *albicans* SC5314 and brains assessed for expression of indicated adhesion molecules by qRT-PCR. Data is analyzed by two-way ANOVA. **P<0.01, ****P<0.001. Data represent mean ± SEM.
(TIFF)

S11 Fig Chemokine production in the brain of uninfected and infected WT and *Card9* ^*-/-*^ mice. Whole brain homogenates were analyzed for Cxcl1, Cxcl2 and Cxcl5 in uninfected and infected WT (filled bars) and *Card9* ^*-/-*^ (empty bars) mice by Luminex array. Mice analyzed at 24 hours were infected with 1.3x10^5^ CFU SC5314, and mice analyzed at 72 hours infected with 7x10^4^ CFU. Data is pooled from two independent experiments and analyzed by two-way ANOVA. *P<0.05, **P<0.01, ***P<0.005, ****P<0.001. n = 6 per time point. Data represent mean ± SEM.
(TIFF)

S12 Fig Purities of cells FACS-sorted from the mouse brain. (**A**) Representative post-sort analysis of neutrophils, microglia and CD45^-^ stromal cells. Left-hand plots are ungated, right-hand plots are gated on CD45^+^ cells. (**B**) Frequency of indicated sorted populations from two independent sorts (WT n = 6, KO n = 6). No post-sort analysis was performed on Ly6C^hi^ monocytes due to insufficient numbers acquired. (**C**) Quantification of *Gfap* and *Mog* transcripts in the CD45^-^ sorted population (WT n = 6, KO n = 6). (**D**) Example CD102/CD31 staining on sorted CD45^-^ cells, gated on CD45- singlets. Plot is representative of 6 sorted populations, from two independent experiments.
(TIFF)

S1 Table Amplification and sequencing primers used for the identification of the *CARD9* c.170G>A mutation. (TIFF)
