# Supplementary figures and images for: CARD9-Dependent Neutrophil Recruitment Protects against Fungal Invasion of the Central Nervous System
Source: PLoS Pathog. 2015 Dec 17;11(12):e1005293. doi: 10.1371/journal.ppat.1005293 (PMC4683065; doi:10.1371/journal.ppat.1005293)

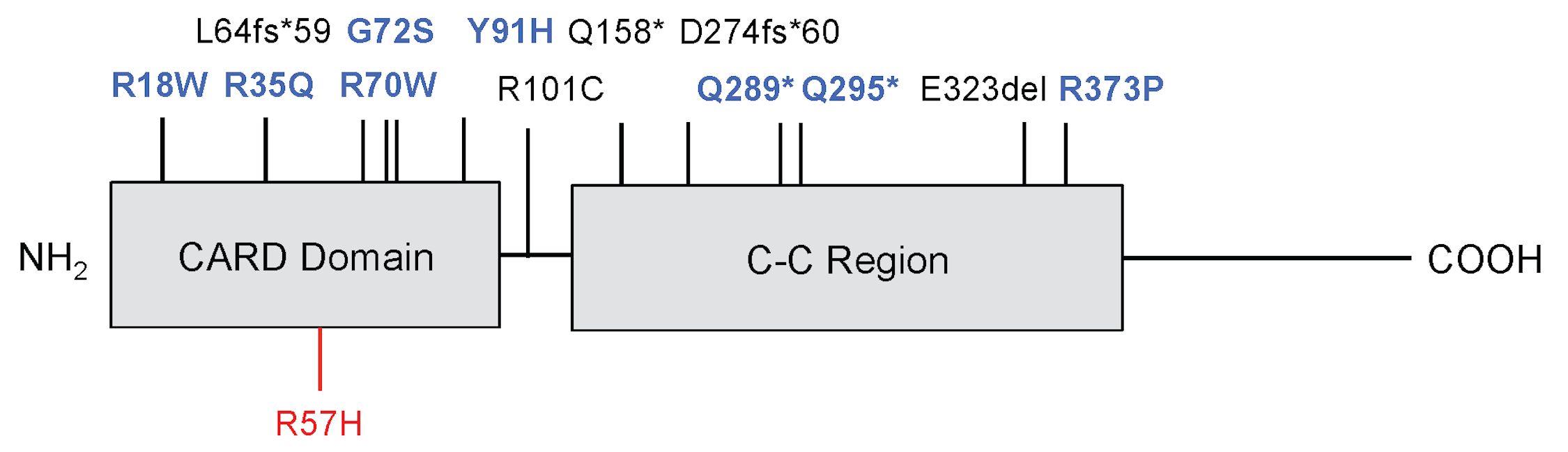

Supplement: S1 Fig — The CARD and coiled-coil (CC) domains are indicated along with reported CARD9 mutations. The R57H mutation of our patient is indicated in red and the previously reported mutations associated with fungal infection of the CNS are indicated in blue. (TIFF) [file ppat.1005293.s001.tiff]

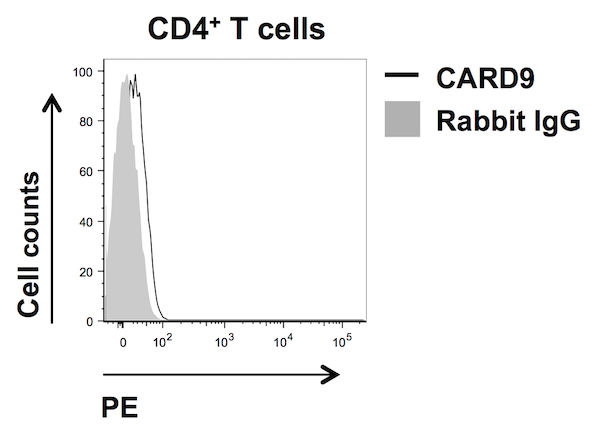

Supplement: S2 Fig — FACS histogram of CARD9 staining in CD4+ T-cells from a healthy donor, compared to the isotype control. (TIFF) [file ppat.1005293.s002.tiff]

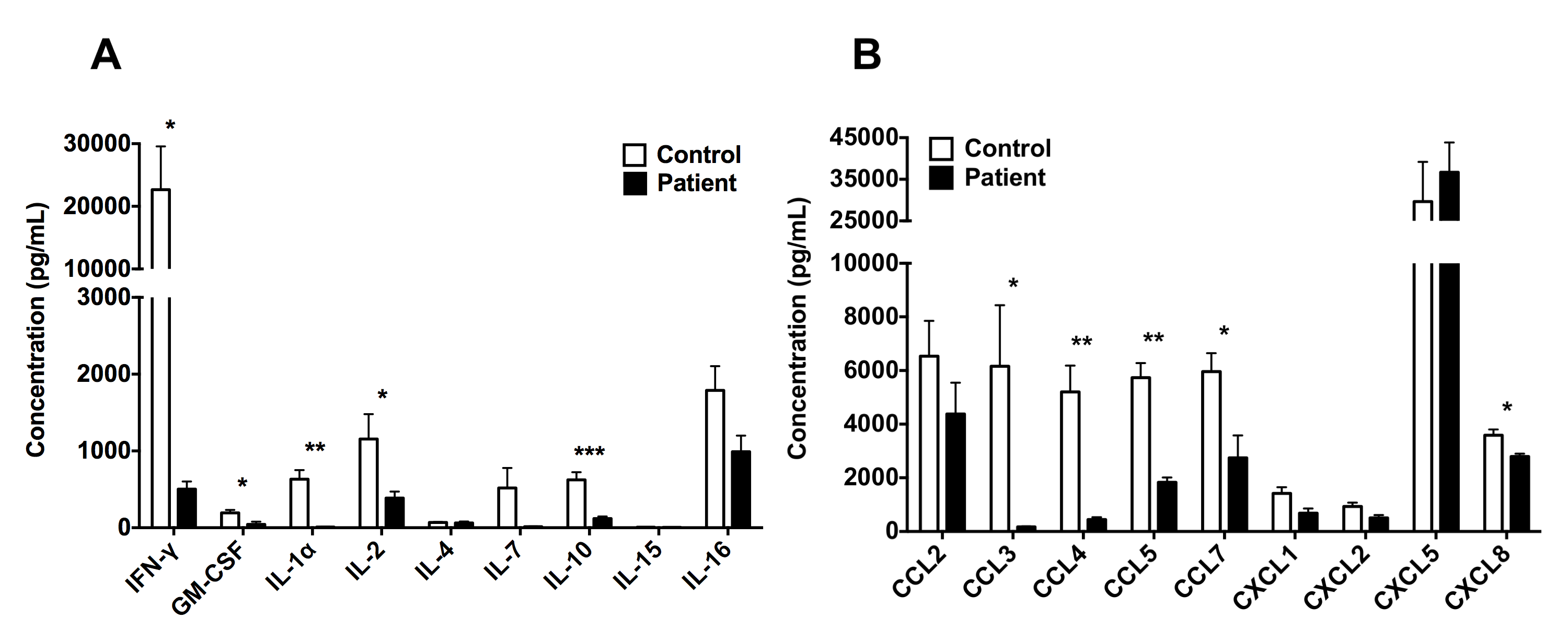

Supplement: S3 Fig — Cytokine (left panel) and chemokine (right panel) production by healthy donor (n = 10) and patient (n = 4) PBMCs after 48 hours of stimulation with heat-killed C. albicans (n = 4–10 independent experiments). Data is analyzed by Mann Whitney U-test or unpaired t-test, where appropriate. *P < 0.05; **P < 0.01; ***P < 0.001; ****P < 0.0001. Data represent mean ± SEM. (TIFF) [file ppat.1005293.s003.tiff]

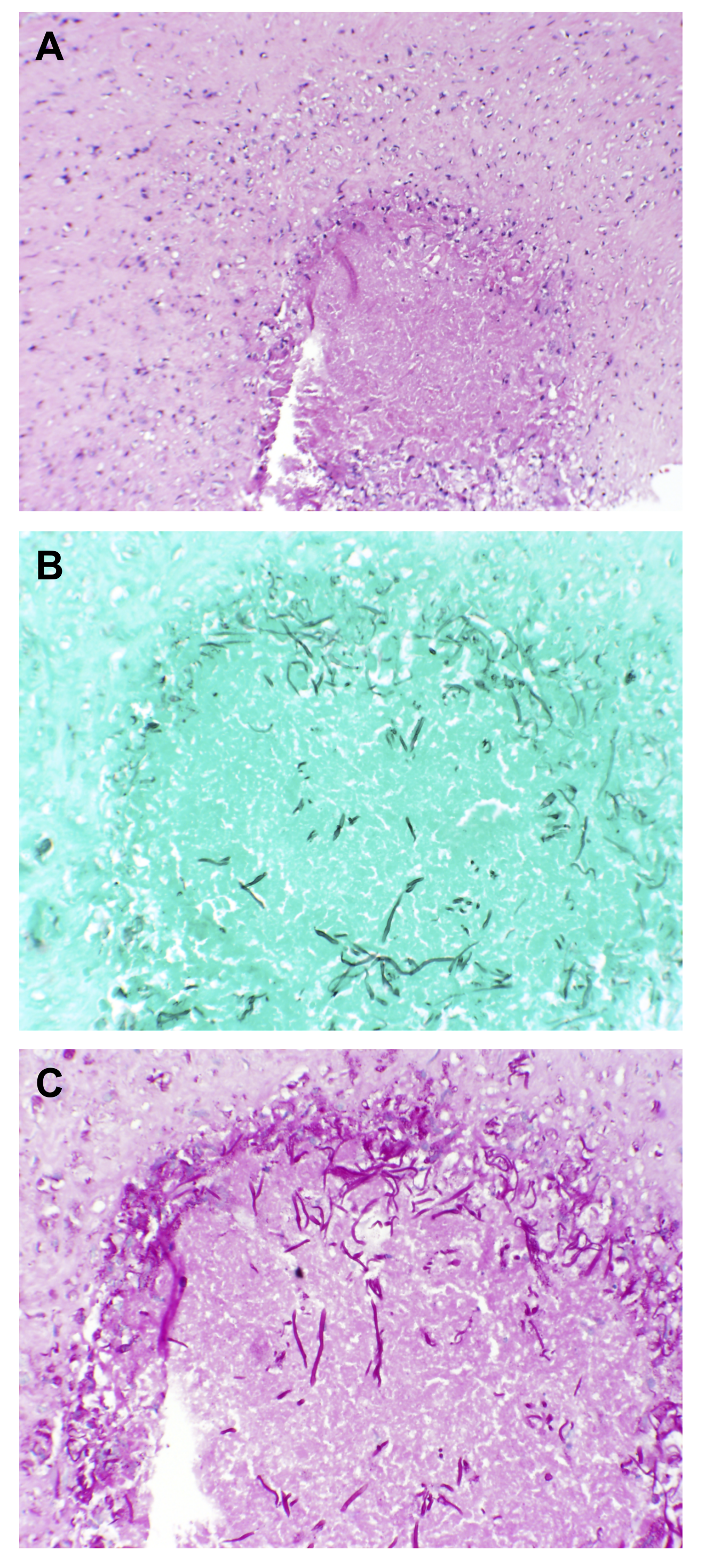

Supplement: S4 Fig — (A) Hematoxylin and eosin (H&E), (B) Grocott-Gomori methenamine-silver (GMS) and (C) Periodic acid-Schiff (PAS) stains of the patient’s brain biopsy sample showing filamentous fungal elements consistent with C. albicans and absence of neutrophil infiltration in the infected tissue (magnification, A, ×100; B-C, ×200). (TIFF) [file ppat.1005293.s004.tiff]

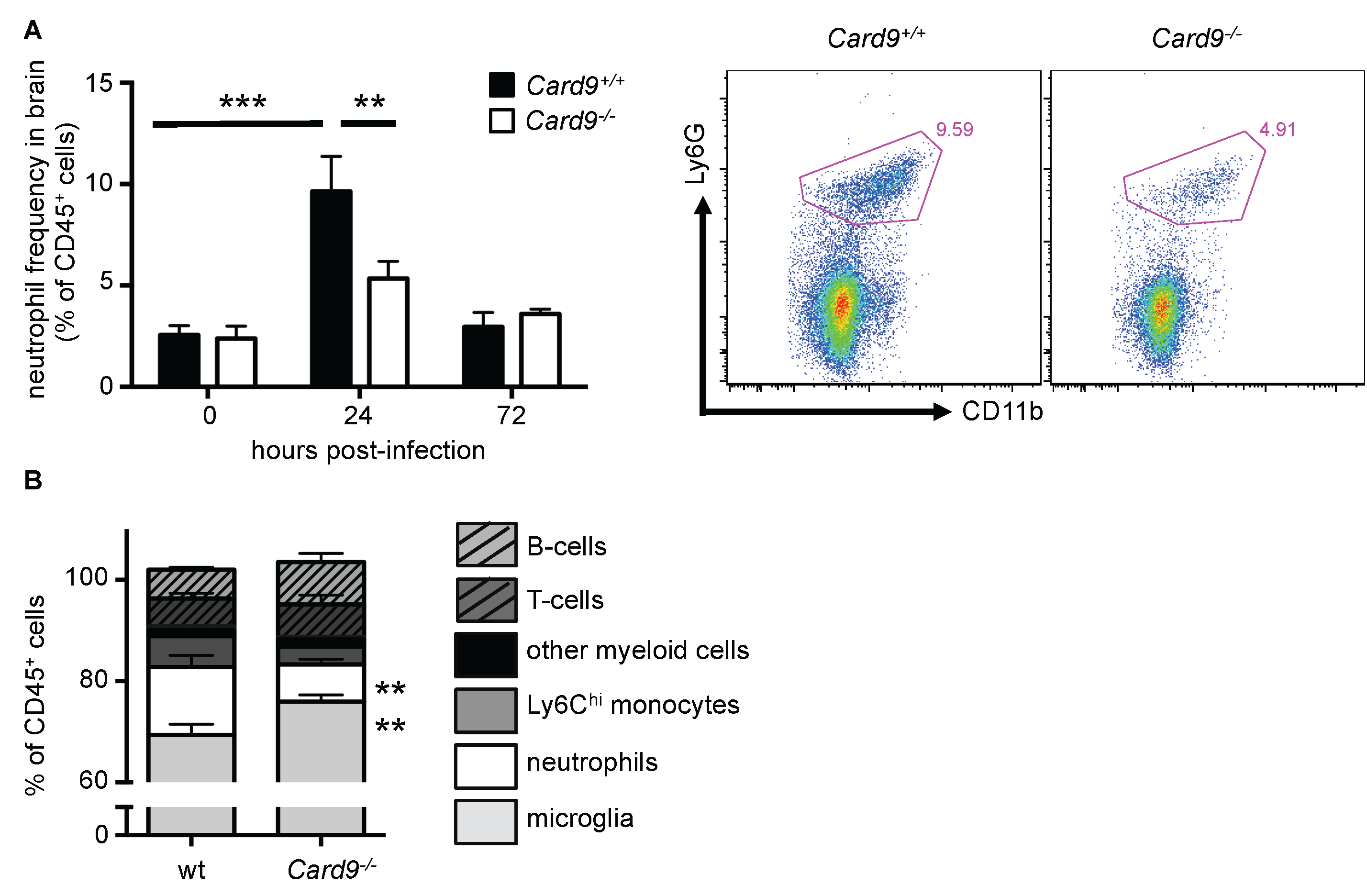

Supplement: S5 Fig — (A) Neutrophil frequency was determined by FACS in the brain of WT (filled bars, 0 hours n = 5, 24 hours n = 6–13, 72 hours n = 6–13) and Card9 -/- (empty bars, 0 hours n = 5–6, 24 hours n = 6–14, 72 hours n = 6–12) mice; data analyzed by two-way ANOVA. Example plots are from 24 hours post-infection and gated on live CD45+ single cells. (B) Relative frequencies of different subsets of hematopoietic CD45+ cells in the brain of WT (n = 6) and Card9 -/- (n = 6) mice at 24 hours post-infection (dose: 1.3x10^5^ CFU) was determined by FACS. ‘Other (myeloid)’ cells represent eosinophils, dendritic cells and macrophages. Data is pooled from two independent experiments. **P<0.01, ***P<0.005 by two-way ANOVA. Data represent mean ± SEM. (TIFF) [file ppat.1005293.s005.tiff]

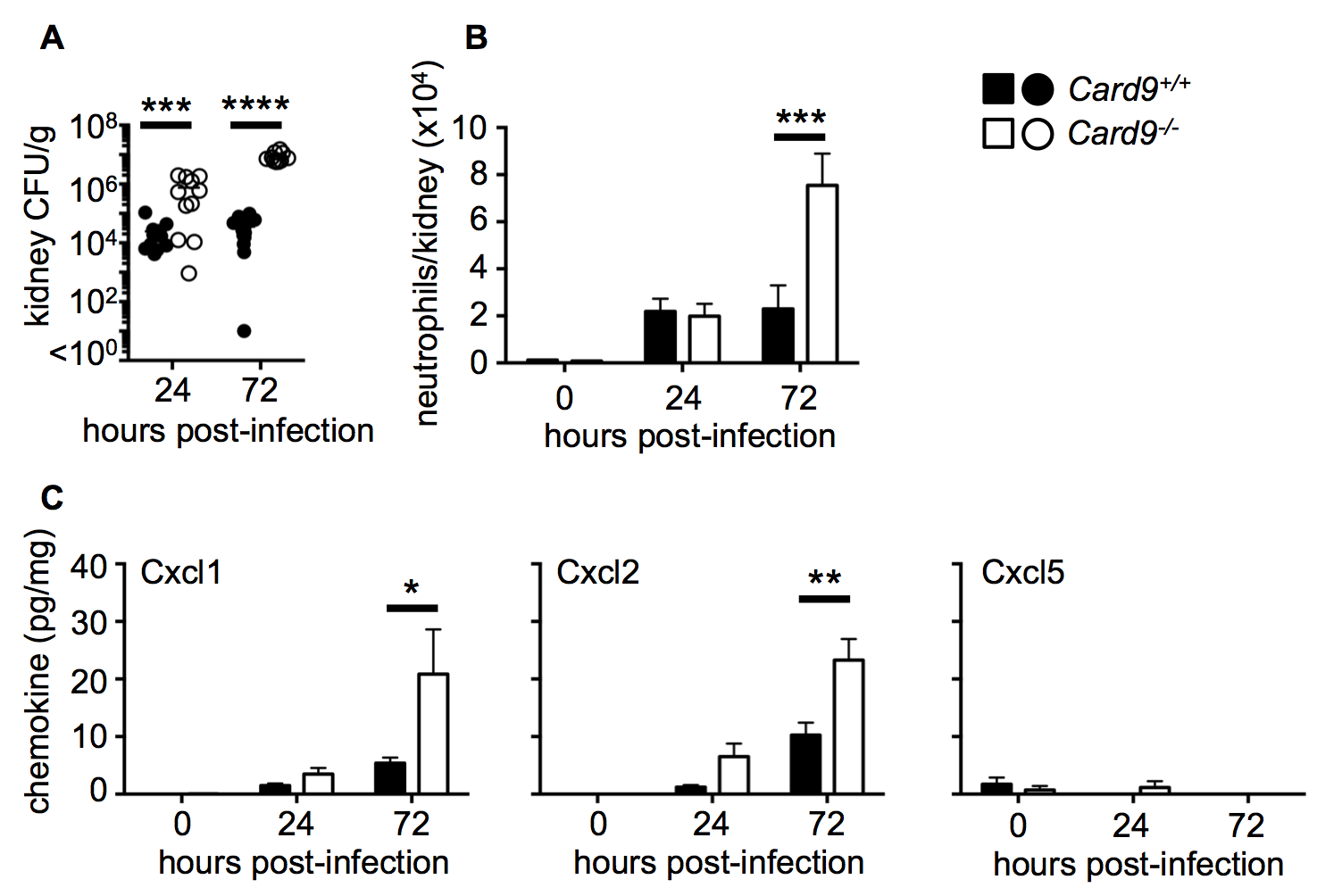

Supplement: S6 Fig — (A) Kidney fungal burdens in WT (filled circles) and Card9 -/- (empty circles) mice at 24 (WT n = 12, KO n = 11) and 72 (WT n = 12, KO n = 10) hours post-infection; data pooled from 3–4 independent experiments and analyzed by Mann Whitney U-test. (B) Neutrophil numbers were determined by FACS in the left kidney of WT (filled bars, n = 6 per time point) and Card9 -/- (empty bars, n = 6 per time point) mice; data analyzed by two-way ANOVA. (C) Whole kidney homogenates were analyzed for Cxcl1, Cxcl2 and Cxcl5 in uninfected and infected WT (filled bars, n = 6 per time point) and Card9 -/- (empty bars, n = 6 per time point) mice by Luminex array. Data is pooled from two independent experiments and analyzed by two-way ANOVA. *P<0.05, **P<0.01, ***P<0.005, ****P<0.001. Data represent mean ± SEM. (TIFF) [file ppat.1005293.s006.tiff]

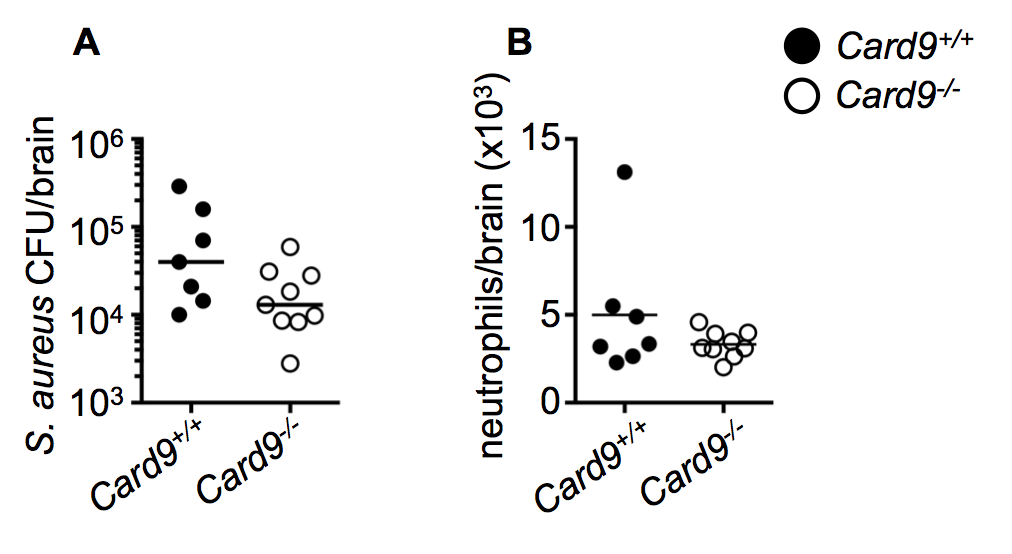

Supplement: S7 Fig — WT (n = 7) and Card9 -/- (n = 9) mice were infected intravenously with 2x10^7^ CFU of S. aureus and euthanized at 48 hours post-infection for (A) bacterial brain burden and (B) accumulation of neutrophils in the brain by FACS. Data is pooled from two independent experiments. (TIFF) [file ppat.1005293.s007.tiff]

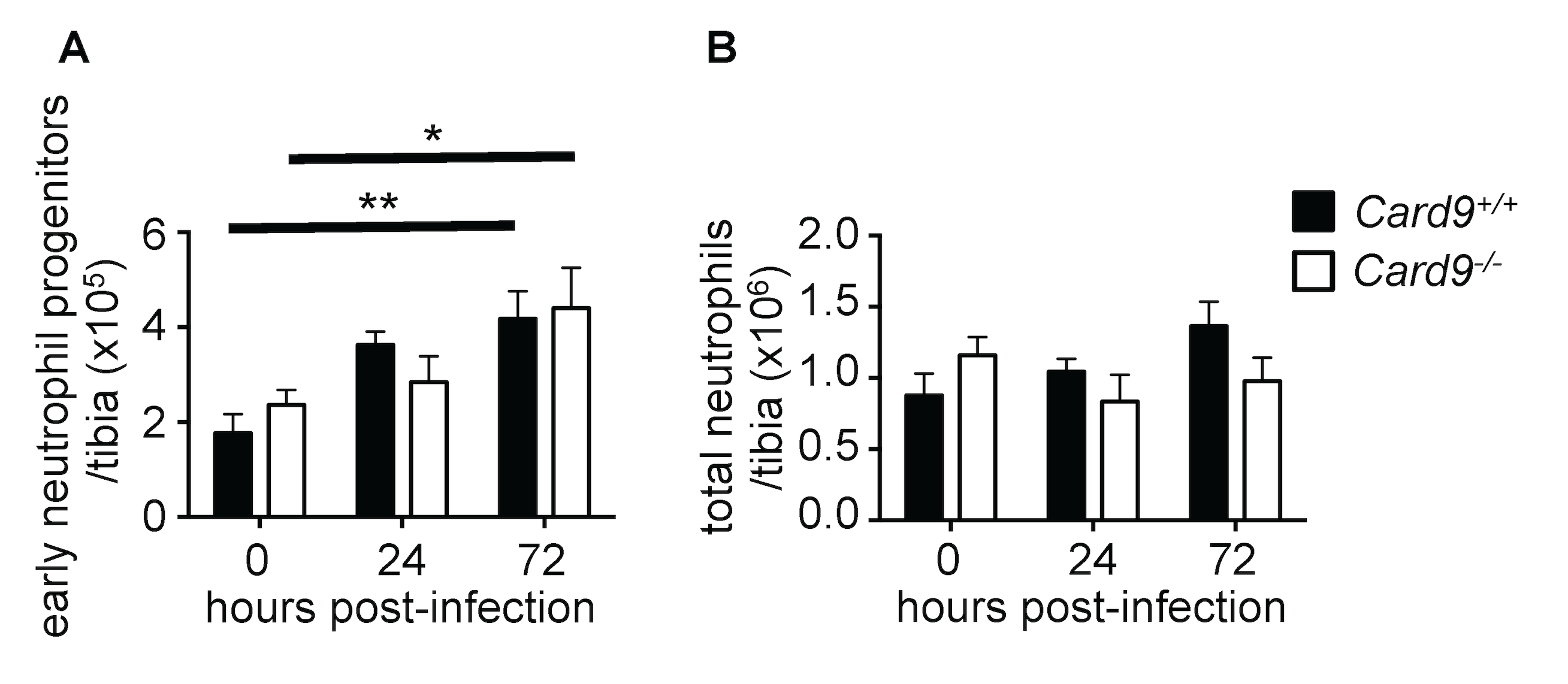

Supplement: S8 Fig — (A) The number of early neutrophil progenitors (Ly6Gint CD11b+) and (B) total neutrophils (Ly6Gint CD11b+ and Ly6Ghi CD11b+ combined) were determined by FACS in the bone marrow of WT (filled bars, 0 hours n = 6, 24 hours n = 6, 72 hours n = 7) and Card9 -/- (empty bars, 0 hours n = 7, 24 hours n = 6, 72 hours n = 6) mice at indicated time points. Mice analyzed at 24 hours were infected with 1.3x10^5^ CFU SC5314, and mice analyzed at 72 hours infected with 7x10^4^ CFU. Data is pooled from 2–4 independent experiments and analyzed by two-way ANOVA. *P<0.05, **P<0.01. Data represent mean ± SEM. (TIFF) [file ppat.1005293.s008.tiff]

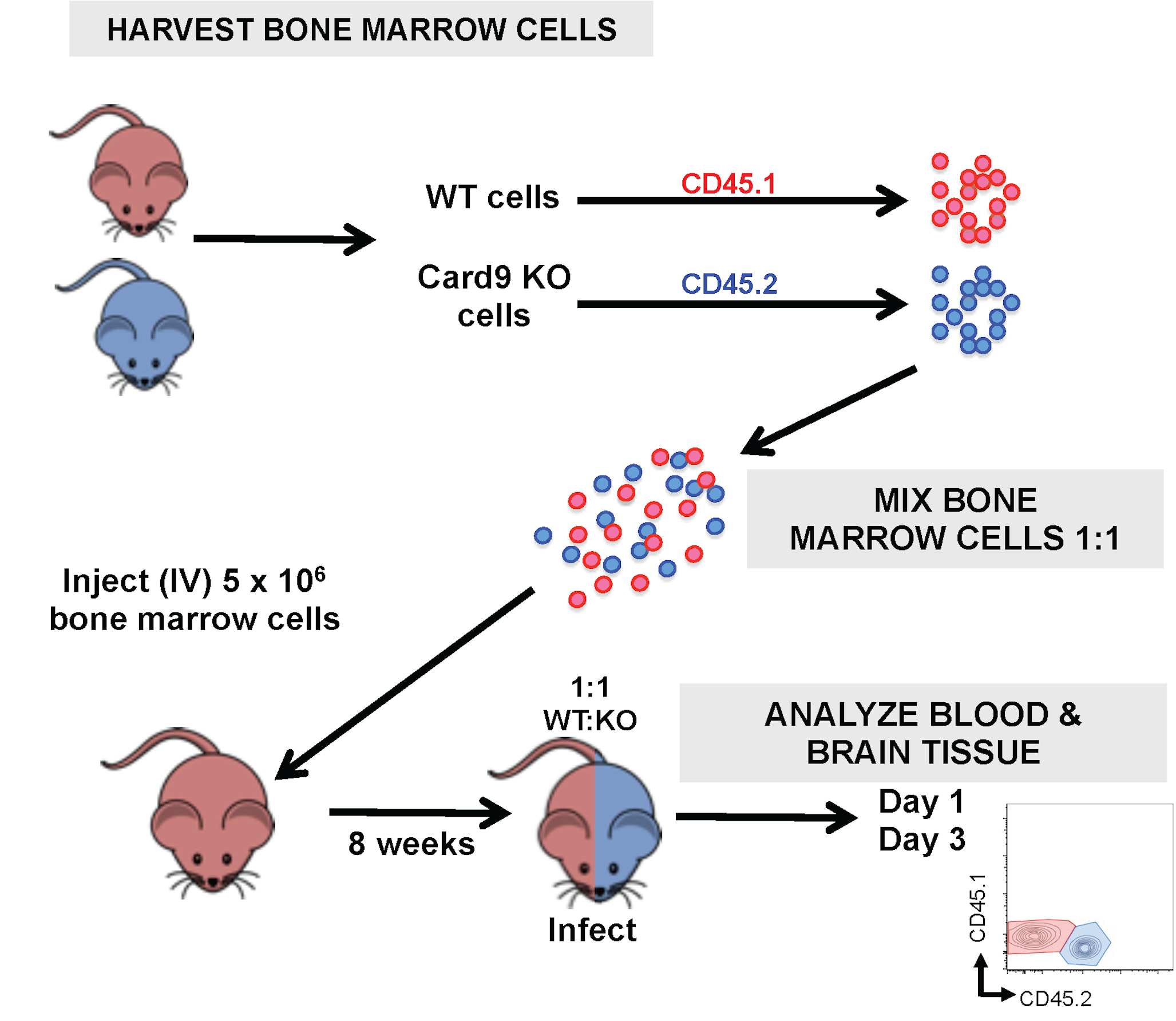

Supplement: S9 Fig — WT CD45.1+ and Card9 -/- CD45.2+ animals were used as donors for the isolation of bone marrow cells that were mixed in a 1:1 ratio prior to transfer. Mixed bone marrow was injected intravenously into irradiated WT CD45.1+ recipient mice, which were left to reconstitute for 8 weeks post-transfer. Chimera status was confirmed by FACS on a sample of peripheral blood prior to infection. (TIFF) [file ppat.1005293.s009.tiff]

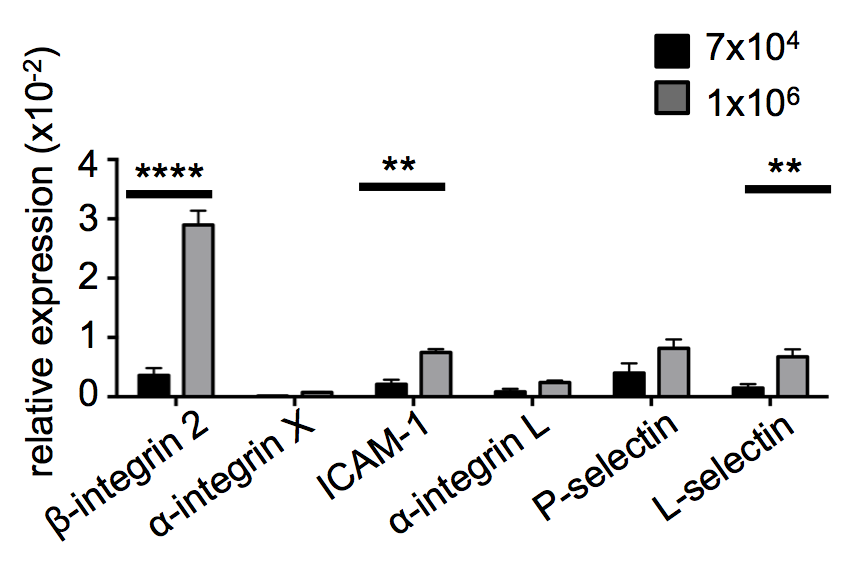

Supplement: S10 Fig — WT animals were systemically infected with a low (7x10^4^) or a high inoculum (1x10^6^) of C. albicans SC5314 and brains assessed for expression of indicated adhesion molecules by qRT-PCR. Data is analyzed by two-way ANOVA. **P<0.01, ****P<0.001. Data represent mean ± SEM. (TIFF) [file ppat.1005293.s010.tiff]

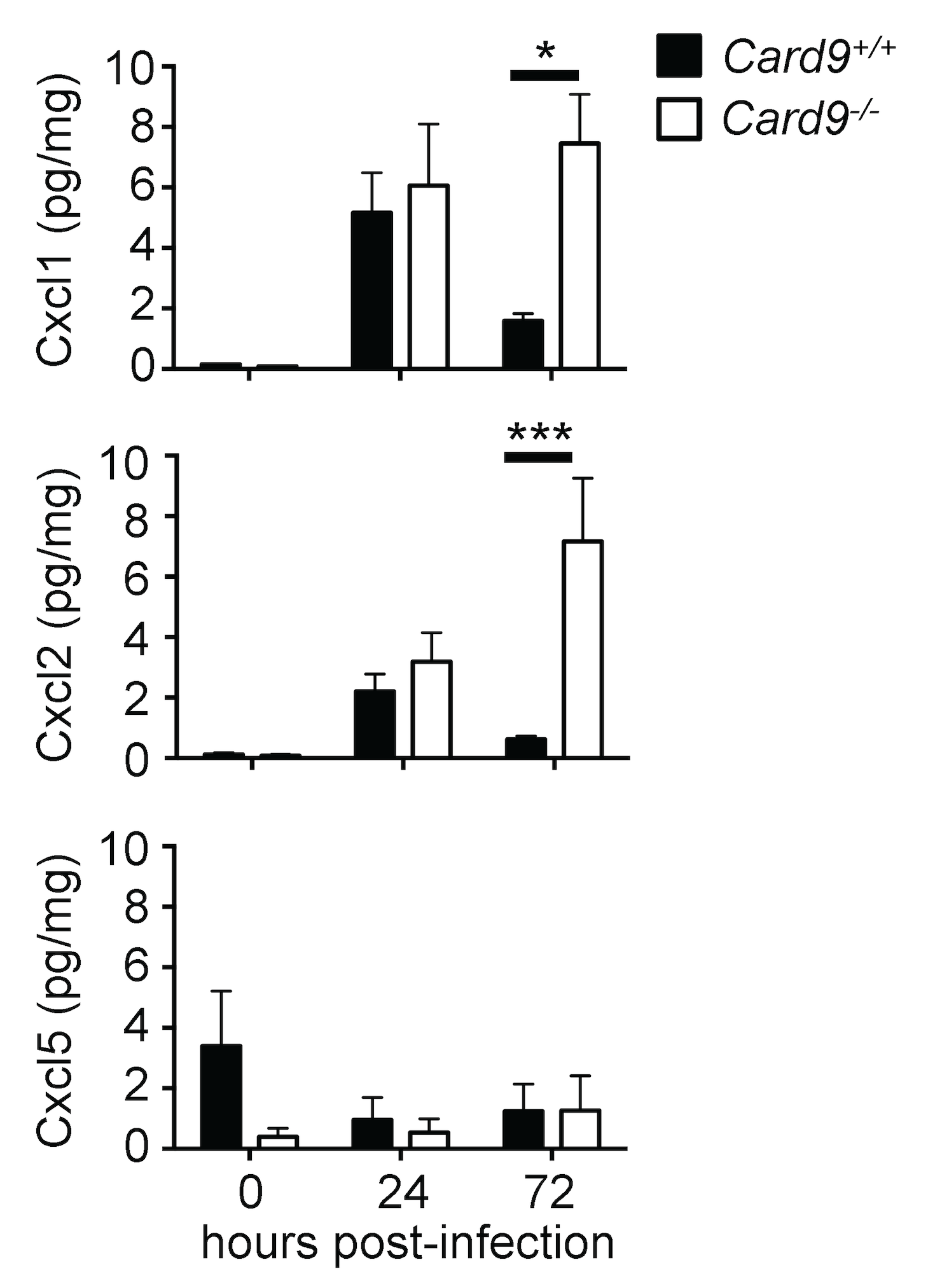

Supplement: S11 Fig — Whole brain homogenates were analyzed for Cxcl1, Cxcl2 and Cxcl5 in uninfected and infected WT (filled bars) and Card9 -/- (empty bars) mice by Luminex array. Mice analyzed at 24 hours were infected with 1.3x10^5^ CFU SC5314, and mice analyzed at 72 hours infected with 7x10^4^ CFU. Data is pooled from two independent experiments and analyzed by two-way ANOVA. *P<0.05, **P<0.01, ***P<0.005, ****P<0.001. n = 6 per time point. Data represent mean ± SEM. (TIFF) [file ppat.1005293.s011.tiff]

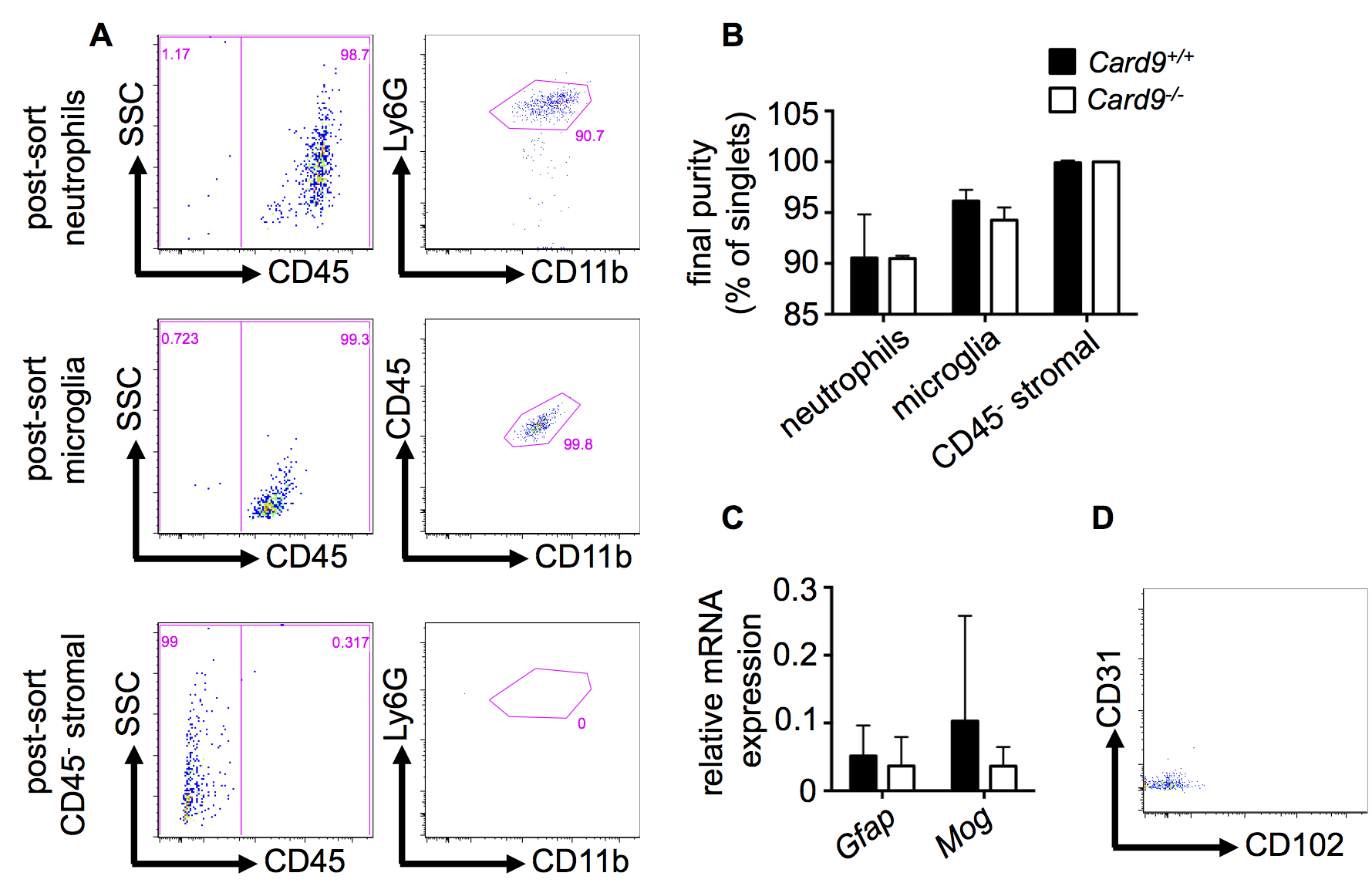

Supplement: S12 Fig — (A) Representative post-sort analysis of neutrophils, microglia and CD45- stromal cells. Left-hand plots are ungated, right-hand plots are gated on CD45+ cells. (B) Frequency of indicated sorted populations from two independent sorts (WT n = 6, KO n = 6). No post-sort analysis was performed on Ly6Chi monocytes due to insufficient numbers acquired. (C) Quantification of Gfap and Mog transcripts in the CD45- sorted population (WT n = 6, KO n = 6). (D) Example CD102/CD31 staining on sorted CD45- cells, gated on CD45- singlets. Plot is representative of 6 sorted populations, from two independent experiments. (TIFF) [file ppat.1005293.s012.tiff]

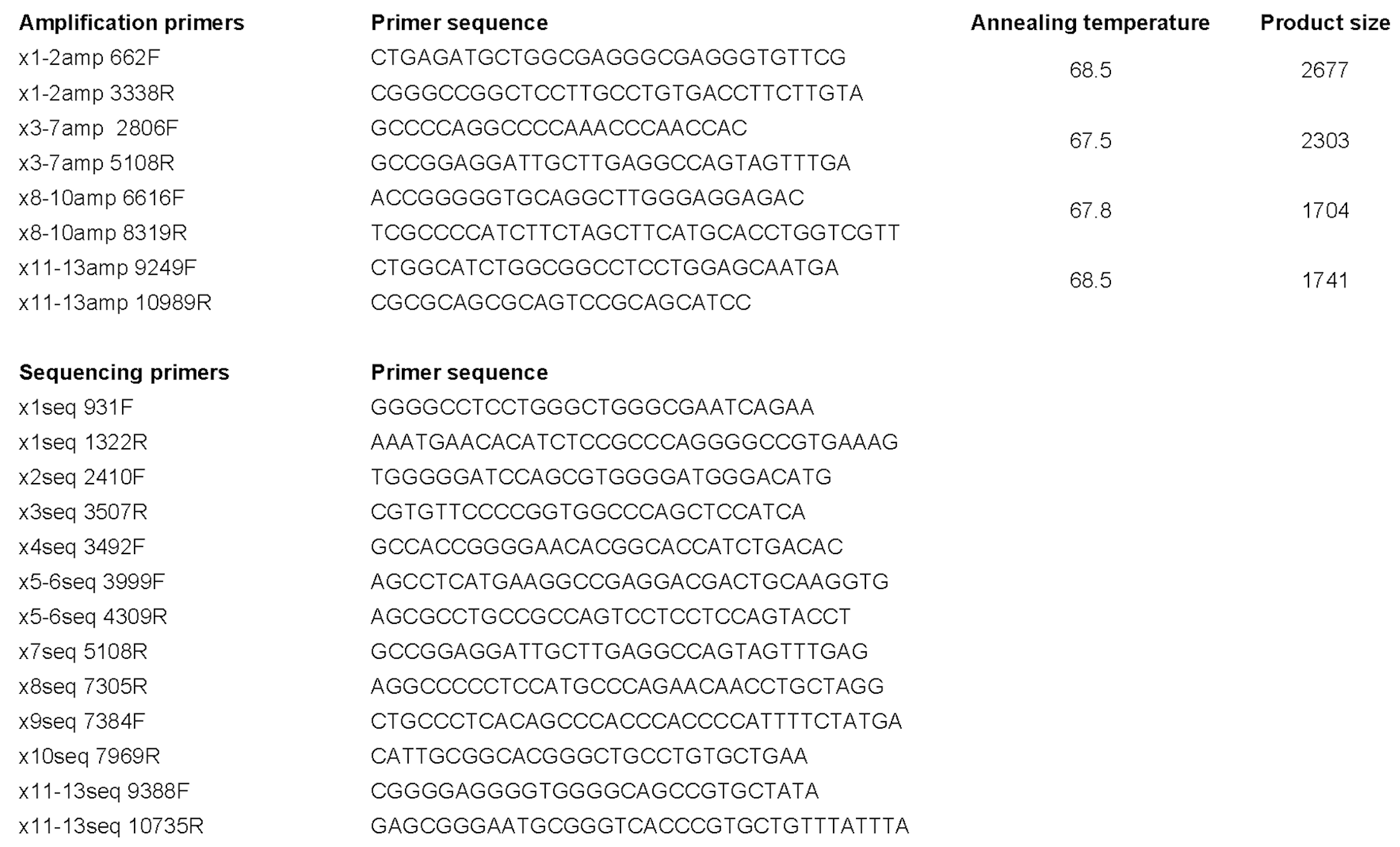

Supplement: S1 Table — (TIFF) [file ppat.1005293.s013.tiff]
